# Long-term running exercise improves cognitive function and promotes microglial glucose metabolism and morphological plasticity in the hippocampus of APP/PS1 mice

**DOI:** 10.1186/s12974-022-02401-5

**Published:** 2022-02-05

**Authors:** Shan-shan Zhang, Lin Zhu, Yan Peng, Lei Zhang, Feng-lei Chao, Lin Jiang, Qian Xiao, Xin Liang, Jing Tang, Hao Yang, Qi He, Yi-jing Guo, Chun-ni Zhou, Yong Tang

**Affiliations:** 1 Department of Histology and Embryology, Faculty of Basic Medical Sciences, Chongqing Medical University, Chongqing, 400016 People’s Republic of China; 2 Laboratory of Stem Cell and Tissue Engineering, Chongqing Medical University, Chongqing, 400016 People’s Republic of China; 3 Experimental Teaching Management Center, Chongqing Medical University, Chongqing, 400016 People’s Republic of China; 4 Department of Radioactive Medicine, Chongqing Medical University, Chongqing, 400016 People’s Republic of China; 5 Department of Pathophysiology, Chongqing Medical University, Chongqing, 400016 People’s Republic of China

**Keywords:** Alzheimer’s disease, Running exercise, Hippocampus, Microglia, TREM2

## Abstract

**Background:**

The role of physical exercise in the prevention of Alzheimer’s disease (AD) has been widely studied. Microglia play an important role in AD. Triggering receptor expressed in myeloid cells 2 (TREM2) is expressed on microglia and is known to mediate microglial metabolic activity and brain glucose metabolism. However, the relationship between brain glucose metabolism and microglial metabolic activity during running exercise in APP/PS1 mice remains unclear.

**Methods:**

Ten-month-old male APP/PS1 mice and wild-type mice were randomly divided into sedentary groups or running groups (AD_Sed, WT_Sed, AD_Run and WT_Run, *n* = 20/group). Running mice had free access to a running wheel for 3 months. Behavioral tests, [18]F-FDG-PET and hippocampal RNA-Seq were performed. The expression levels of microglial glucose transporter (GLUT5), TREM2, soluble TREM2 (sTREM2), TYRO protein tyrosine kinase binding protein (TYROBP), secreted phosphoprotein 1 (SPP1), and phosphorylated spleen tyrosine kinase (p-SYK) were estimated by western blot or ELISA. Immunohistochemistry, stereological methods and immunofluorescence were used to investigate the morphology, proliferation and activity of microglia.

**Results:**

Long-term voluntary running significantly improved cognitive function in APP/PS1 mice. Although there were few differentially expressed genes (DEGs), gene set enrichment analysis (GSEA) showed enriched glycometabolic pathways in APP/PS1 running mice. Running exercise increased FDG uptake in the hippocampus of APP/PS1 mice, as well as the protein expression of GLUT5, TREM2, SPP1 and p-SYK. The level of sTREM2 decreased in the plasma of APP/PS1 running mice. The number of microglia, the length and endpoints of microglial processes, and the ratio of GLUT5^+^/IBA1^+^ microglia were increased in the dentate gyrus (DG) of APP/PS1 running mice. Running exercise did not alter the number of 5-bromo-2′-deoxyuridine (BrdU)^+^/IBA1^+^ microglia but reduced the immunoactivity of CD68 in the hippocampus of APP/PS1 mice.

**Conclusions:**

Running exercise inhibited TREM2 shedding and maintained TREM2 protein levels, which were accompanied by the promotion of brain glucose metabolism, microglial glucose metabolism and morphological plasticity in the hippocampus of AD mice. Microglia might be a structural target responsible for the benefits of running exercise in AD. Promoting microglial glucose metabolism and morphological plasticity modulated by TREM2 might be a novel strategy for AD treatment.

**Supplementary Information:**

The online version contains supplementary material available at 10.1186/s12974-022-02401-5.

## Introduction

Alzheimer's disease (AD), the most common cause of dementia, has become an important public issue worldwide and results in progressive cognitive decline and disability [1]. The β-amyloid (Aβ) hypothesis and Tau hypothesis have gradually become the dominant theories in AD research [2]. However, recent clinical trials involving new drugs targeting these theories have failed to delay memory and cognitive decline in patients with AD [3]. Therefore, it is urgent to find new AD treatments.

The benefits of physical exercise on cognitive function are well recognized, and the utilization of physical exercise to prevent AD has been widely advocated [4]. Many clinical studies have confirmed that running exercise can alleviate memory loss in AD patients [5, 6]. In addition, long-term running exercise has been demonstrated to improve learning and memory in AD model mice [7, 8]. However, the mechanism by which running exercise improves cognitive decline in AD remains to be further studied.

It has been reported that Aβ deposition alone does not lead to cognitive impairment, but local decreased brain glucose metabolism combined with Aβ deposition results in subsequent cognitive decline in patients [9]. [18]F-Fluoro-2-deoxy-d-glucose/positron emission tomography ([18]F-FDG-PET) can indicate glucose metabolism and has been recognized to assist in neurodegenerative disease diagnosis [10]. A decrease in regional FDG uptake in the brains of AD patients has also been reported [11]. Moreover, the standard uptake value (SUV) of FDG has been reported to be significantly reduced in the cerebra of 13-month-old 5xFAD mice [12] and 15-month-old APP/PS1 mice [13]. Physical exercise, as an important physiological stimulus, can promote cellular glucose uptake and energy metabolism [14] by activating AMP-activated protein kinase (AMPK) [15] and mammalian target of rapamycin (mTOR) [16]. Shimada et al. found that 3 months of aerobic exercise increased FDG uptake in the left posterior entorhinal cortex, left superior temporal gyrus, and right superior temporal polar gyrus in elderly individuals [17]. Obisesan et al. found that 6 months of aerobic training increased glucose metabolism in the hippocampus of elderly patients with mild cognitive impairment (MCI) [18]. However, it is not clear whether long-term running exercise affects regional brain glucose metabolism in patients with AD or what cellular mechanisms are involved. FDG-PET is commonly thought to reflect neuronal plasticity [19] or astrocyte glucose uptake [20]. However, the effects of microglial glucose metabolism on brain metabolism have only recently been discovered [21]. Microglial activation depends on high metabolic activity [22], and this activation has become an important consumer of glucose metabolism in the AD brain [23]. However, it is unclear whether microglia are involved in the improvement in regional brain glucose metabolism after running exercise.

If microglial glucose metabolism is involved in the elevated brain glucose metabolism induced by running exercise, what might be the underlying molecular mechanism? Triggering receptor expressed in myeloid cells 2 (TREM2) is a member of the immunoglobulin/lectin-like receptor superfamily that is highly expressed on microglia [24]. TREM2 mediates intracellular signal transduction through its adaptor protein DNAX-activation protein 12 (DAP12), also known as TYROBP, which contains an immunoreceptor tyrosine-based activation motif (ITAM) [25]. Studies have reported that TREM2 triggers DAP12 by binding to a TREM2 ligand (TREM2-L), such as Aβ or apolipoprotein E (APOE) [26, 27], and then promoting SYK (Tyr525/526) phosphorylation, which leads to phagocytosis and degradation [28, 29]. TREM2 has been highlighted to regulate microglial metabolism in AD [30], and TREM2 loss-of-function mutations, such as R47H and T66M, are associated with a high risk of AD [31]. Piers et al. found significant metabolic defects in microglia of AD patients with the TREM2 variant, including decreased mitochondrial respiratory flux and glycolytic immune metabolic switch failure [32]. Ulland et al. confirmed that TREM2 maintained the metabolic activity of microglia through mTOR signaling activation, which supports long-term microglial survival, proliferation, and phagocytosis [33]. In addition, it was reported that translocation protein 18 kDa (TSPO) signaling, a radioactive indicator of microglial activity [34], was reduced in TREM2^−/−^ mice and TREM2 T66M mutant mice, and FDG uptake was decreased in TREM2^−/−^ mice and homozygous TREM2 p-T66M knock-in mice [35, 36], suggesting that TREM2-dependent microglial metabolic activity is associated with brain glucose metabolism. However, whether running exercise affects TREM2-related microglial metabolic activity and is related to brain metabolism remains to be further studied. Of note, the extracellular domain of TREM2 can be shed by a disintegrin and metallopeptidase (ADAM) 10/17 to become soluble TREM2 (sTREM2) [37]. Clinical studies have found significant increases in sTREM2 levels in the cerebrospinal fluid (CSF) of AD patients [38, 39]. The effects of running exercise on both TREM2 and sTREM2 in AD need to be further investigated.

In this study, 10-month-old male APP/PS1 mice were subjected to long-term voluntary wheel running for 3 months to determine its therapeutic effects on cognitive impairment in an AD model. The potential mechanism by which long-term running exercise improves cognitive function was explored using RNA sequencing, [18]F-FDG-PET, immunohistochemistry, stereological methods, immunofluorescence, qRT–PCR, western blotting and ELISA. This study aimed to investigate the beneficial effects of long-term running exercise on hippocampal and microglial glucose metabolism and morphological plasticity of microglia in APP/PS1 mice with the hope of finding a new target to improve cognition in AD.

## Methods

### Animals

APPswe/PSEN1dE9 mice were obtained from the Animal Model Institute of Nanjing University, China. To exclude the influence of sex differences on cognitive impairment in AD [40], only male mice were used in this study. Forty 10-month-old male APP/PS1 mice and forty wild-type littermates were randomly divided into sedentary groups (WT_Sed and AD_Sed, *n* = 20/group) and running groups (WT_Run and AD_Run, *n* = 20/group). Single housing had an impact on social behavior [41]. To minimize the influence of a single housing on the results, each mouse from each group was single caged in standard polypropylene cages (290 × 180 × 160 mm) throughout the experiment. Each running mouse had free access to a running wheel (12 cm in diameter) for 3 months, which was connected to a counter to record the running distance (Wuhan Yihong Technology, China). Body weight was measured weekly throughout the study. Mice were intraperitoneally injected with 5-bromo-2′-deoxyuridine (BrdU) (10 mg/kg; Sigma) for 7 days and sacrificed 28 days after the last injection [42]. Mice were housed at 22 ± 1 °C with 55 ± 5% humidity on a 12-h light–dark cycle with ad libitum access to water and food. All animal procedures followed double-blind principles and the National Institutes of Health Guide for the Care and Use of Laboratory Animals (NIH Publication No. 85–23).

### Behavioral testing

New object recognition (NOR) [43] was performed in a 30 × 30 × 45 cm^3^ open box. All mice at the age of 13 months were placed in the center of the box to acclimate for 10 min per day for 3 days. On the test day, mice were first placed in the box for 10 min with two identical objects and then placed in the box again for 10 min with one of the objects, which was replaced with a new object 1 h later. The time exploring the object (nose tip toward the object at a distance ≤ 2 cm) was recorded; exploration times of less than 8 s were excluded [44]. The discrimination index (DI; DI = total time spent with a new object/total time spent with two objects) was calculated.

The Morris water maze (MWM) [45] was used to assess spatial learning and memory and utilized a circular pool (120 cm in diameter) and a platform (10 cm in diameter) divided into four quadrants filled with white water (22–25 ℃). In the positioning navigation experiment (days 1–6), the platform was fixed in a quadrant and hidden 1 cm below the water. Each mouse was placed on the platform for 15 s of brief training and then placed into a quadrant of water facing the pool wall to find the platform. If the platform was not found within 60 s, the mouse was guided to the platform for another 15 s of training. Each mouse was tested in all four quadrants in a random order once each day in each quadrant. In the space exploration experiment on day 7, the platform was removed, and the mice were placed in two different positions away from the platform and allowed to swim for 60 s. A video tracking system recorded each mouse's swimming speed, escape latency and frequency across the platform.

### [18]F-FDG-PET

Five mice in each group were given PET scans at the age of 10 months and 13 months. After fasting for 12 h, mice were anesthetized with isoflurane (4% for induction, 2% for maintenance) supplemented with oxygen, and [18]F-FDG was intraperitoneally injected. The injection dose and body weight of each mouse were recorded. Approximately 40 min later, the mice were anesthetized again and scanned using a nanoScan PET/MRI system (Mediso, Hungary). During scanning, mice were placed on a 37 ℃ constant temperature bed, and their respiratory rates were continuously monitored. MRI-based attenuation correction PET images were reconstructed with Nucline software (Bioscan, USA). PET images were matched to a predefined mouse brain atlas template (Additional file 1: Fig. S1a), and the standardized uptake value (SUV) of the volume of interest (VOI) was calculated for semiquantitative analysis using POMD v.3.4 (PMOD Technologies, Switzerland) [SUV = VOI activity concentration (Bq/cm)/(injected dose (Bq)/body weight (g))].

### RNA-Seq

Mice were intraperitoneally anesthetized with 1% pentobarbital (Sigma). The brains of mice (three mice in each of the following groups: WT_Sed, AD_Sed, and AD_Run) were directly dissected, and the hippocampus was isolated on ice and soaked in RNA protect tissue reagent (QIAGEN). Total RNA was extracted with TRIzol (Invitrogen). Ribosomal RNA (rRNA) was removed, and the RNA was fragmented into small pieces. The cleaved RNA fragments were copied into first-strand cDNA followed by second-strand cDNA synthesis. This process removes the RNA template and synthesizes a replacement strand, incorporating dUTP in place of dTTP to generate ds cDNA. The incorporation of dUTP quenches the second strand during amplification. After quality and quantity evaluation, the cDNA library was created, and paired-end sequencing was performed on a BGISEQ-500/MGISEQ-2000 System (BGI-Shenzhen; Beijing Genomic Institute in Shenzhen, China). Raw data were filtered with SOAPnuke v.1.5.2. The clean reads were mapped to the reference genome using HISAT2 v.2.0.4. Bowtie2 v.2.2.5 was applied to align the clean reads to the gene set, a database built by BGI-Shenzhen, and the expression levels of the genes were calculated with RSEM v.1.2.12. Differential expression analysis was performed using DESeq2 v.1.4.5 [46]. Gene set enrichment analysis (GSEA) was performed using GSEA v.4.1.0 [47].

### Immunohistochemistry

Five mice in each group were transcardially perfused with saline containing heparin followed by 4% paraformaldehyde solution. The brains were removed and divided into two hemispheres, fixed with paraformaldehyde and preserved at 4 °C. The brains were dehydrated with sucrose solutions (10%, 20%, and 30%) in 0.1 M phosphate-buffered saline (PBS), embedded in optimal cutting temperature (OCT) compound, and quickly frozen for 10 min. One hemisphere of the brain was cut into 50 μm thick sections, and another hemisphere was cut into 30 μm thick sections using a frozen slicer (Leica, Germany). All 50 μm sections containing the hippocampus were randomly divided into 5 equal series starting from the first section (*n* = 12–14 sections/each series), and the 30 μm sequences were divided into 20 equal series (*n* = 4–5 sections/each series) and stored in alcohol at − 20 ℃.

One series of 50 μm sections was washed twice with 0.01 M PBS and permeabilized in PBS containing 0.3% Triton X-100/0.1% Tween-20 (PBST) for 1 h, incubated with 0.3% H_2_O_2_ for 20 min to eliminate endogenous peroxidase activity, and blocked in PBST with 10% goat serum/1% fetal serum at 37 °C for 2 h after heat-induced antigen retrieval in citrate buffer for 30 min. Then, the sections were incubated with rabbit anti-IBA1 antibody (Abcam, ab153696, 1:1000) at 4 °C for 48 h, followed by incubation with biotin-labeled goat anti-rabbit IgG and horseradish peroxidase-labeled working solution (ZSDB-BIO, China) at 37 °C for 2 h. Next, sections were visualized with 3,3-diaminobenzidine (DAB), mounted on slides, counterstained with hematoxylin, dehydrated with gradient alcohol series (75%, 80%, 95%, 100%), cleared with xylene, sealed with neutral gum and coverslipped.

### Stereological analysis

The total number of microglia in the hippocampal DG, CA1, and CA3 regions was determined via the optical fractionator method using a morphometry system consisting of a microscope with a camera (Olympus, Japan), a high-precision microcator (ProScan, UK), and stereological analysis software (New CAST, Denmark). Stereological counting and analysis were performed by a double-blind procedure. Images were captured under a × 4 lens. Outlines of the DG, CA1, and CA3 regions were delineated according to *The Mouse Brain in Stereotaxic Coordinates* [48]. The stereological probe (counting point) was superimposed onto the image, and the volume of the region of interest was calculated using Cavalieri's principle [49]. Under the × 100 oil lens, the unbiased counting frame was randomly and equidistantly placed in the delineated area of interest: the area sampling fraction (asf) was 8%, the counting height was 15 μm and the guard height was 3 μm. The number of positive cells in each counting frame was counted successively: the *z*-axis position of the first focused microglia was set as 0 μm, and counting began when the *z*-axis moved below the guard height to 18 μm. The actual thickness (*t*) was recorded, and the overall average sampling fraction of each region was calculated (asf = 15 μm/*t*). The counted number was summed (Σ*Q*^−^), and the total number was calculated: *N* = Σ*Q*^−^ × (1/5) × (1/asf) × (1/tsf) [50].

### Immunofluorescence

One series of the 30 μm sections mentioned above was permeabilized, subjected to antigen retrieval, blocked and then incubated with the following primary antibodies at 4 °C for 36 h: mouse anti-GLUT5 (Santa Cruz, sc-271055, 1:100), mouse anti-TREM2 (Santa Cruz, sc-373828, 1:100), mouse anti-CD68 (Abcam, ab955, 1:500), rabbit anti-IBA1 (Abcam, ab153696, 1:500) and rabbit anti-PSD95 (CST, 3450, 1:500) followed by incubation with the corresponding fluorescent secondary antibodies (1:200) at 37 °C for 2 h: DyLight-549 goat anti-mouse IgG and DyLight-488 goat anti-rabbit IgG. Sections were stained with DAPI and sealed with an anti-fluorescence quencher.

For BrdU staining, after blocking, the sections were incubated with primary antibodies at 4 °C for 36 h: rabbit anti-IBA1 (Abcam, ab153696, 1:500) and chicken anti-NeuN (Sigma, ABN91, 1:400). Sections were treated with 2 M HCl for 50 min, washed with 0.1 M sodium tetraborate buffer and incubated with rat anti-BrdU antibody (Abcam, ab6326, 1:300) at 4 °C for 24 h. Then, the sections were incubated with DyLight-549 goat anti-mouse IgG, Alexa Fluor 488 goat anti-chicken IgG, and DyLight-649 goat anti-rat IgG at 37 °C for 2 h and sealed. For thioflavin S staining, sections were immersed in 50% alcohol containing 0.03% thioflavin S (Sigma) at room temperature in the dark for 10 min, cleaned with 50% alcohol 3 times, and sealed with an anti-fluorescence quencher.

Images were captured using a confocal microscope (Nikon) under a × 60 oil lens and a superresolution microscope system (Olympus) under a × 20 lens. The image threshold was uniformly set using ImageJ v.6.4. “Skeleton analysis” was used to measure the morphology of the microglia [51]. The number of positive cells was determined by “particle analysis” [52], and the number of colabeled cells was counted manually with a double-blind procedure. 3D images were observed and masked using Imaris v.9.0 [53].

### Enzyme-linked immunosorbent assay (ELISA)

The hearts of ten mice in each group were exposed, and blood was drawn from the right atrium into an anticoagulant tube containing heparin and centrifuged for 15 min at 3000×*g*. The plasma was extracted and stored at − 80 °C. The level of sTREM2 in plasma was determined with a mouse sTREM2 ELISA kit **(**J&L, JL20435, China).

The hippocampus of twelve mice in each group was isolated, snap-frozen in liquid nitrogen, and stored at − 80 °C for protein extraction. Hippocampal tissue was added to 8 volumes of RIPA lysis buffer containing 1% phenylmethanesulfonyl fluoride (PMSF) and phosphatase inhibitor (Beyotime, China), homogenized with an ultrasonic processor, and centrifuged at 12,000×*g* for 10 min for supernatant extraction. The protein content was determined with a BCA kit (Beyotime, China), adjusted to a uniform concentration of 10 mg/ml, and stored at − 80 °C. These protein solutions from nine mice in each group were used to detect the levels of TREM2 and sTREM2 in the hippocampus with a mouse TREM2 ELISA kit (EIAab, E12753m, China) and a mouse sTREM2 ELISA kit (J&L, JL20435, China).

### Western blotting

Protein solutions from three mice in each group were denatured with 5 × loading buffer at 95 °C for 10 min and stored at − 20 °C. Twenty to forty micrograms of total protein sample were separated by SDS-polyacrylamide gel electrophoresis using a Bio–Rad protein assay and transferred onto polyvinylidene fluoride (PVDF) membranes. Membranes were blocked in Tris-buffered saline/0.1% Tween buffer (TBST; 25 mM Tris–Cl, 125 mM NaCl, 0.1% Tween-20) with 5% skim milk powder at room temperature for 2 h and incubated with primary antibodies at 4 °C overnight: mouse anti-GLUT5 (Santa Cruz, sc-271055, 1:300), mouse anti-GLUT3 (Abcam, ab150299, 1:1000), mouse anti-ADAM10 (Santa Cruz, sc-28358, 1:500), mouse anti-SYK (Santa Cruz, sc-1240, 1:300), rabbit anti-phospho-SYK (Tyr525/526) (CST, 2710, 1:300), mouse anti-β-tubulin and mouse anti-β-actin (Abmart, 1:1000). The corresponding secondary antibody was incubated at room temperature for 2 h (1:4000). The results were analyzed using SuperSignal West Pico Chemiluminescent Substrate (Thermo Fisher Scientific, USA).

### Quantitative real-time reverse transcription PCR (qRT-PCR)

Total RNA was extracted with TRIzol (Invitrogen). cDNA was synthesized using the PrimeScript RT reagent Kit (Takara) in a reaction of 1 μg of total RNA per reaction. Relative mRNA levels were quantified by qRT–PCR using the SYBR® Green Premix Pro Taq HS qPCR Kit (Accurate Biology, China) with the following specific primers: Trem2-F: CCAAGGAATCAAGAGACC, Trem2-R: CAGTGAGGATCTGAAGTTG; Tyrobp-F: TGCGACTGTTCTTCCGTGAG, Tyrobp-R: TTCCGCTGTCCCTTGACCT; Cd68-F: AGGCTTTTCATTTCCTCTTCCA, Cd68-R: TCCTCTGTTCCTTGGGCTAT; Cd74-F: CGACCTCATCTCTAACCA, Cd74-R: GTACAGGAAGTAAGCAGTG; Itgax-F: ACTGTTCACCACCCAAAGTG, Itgax-R: TGTCCCCTTGTTTTCTCCCA; Spp1-F: TTTCTGATGAACAGTATC, Spp1-R: GAAGATGAACTCTCTAAT.

### Statistical analysis

The results are presented as the mean ± SD, and outliers were excluded if their value exceeded the mean ± 2 SD. All statistical analyses were performed with SPSS v.26.0. Repeated-measures analysis of variance (ANOVA) was used to analyze part of MWM data. Other data were analyzed using one-way ANOVA followed by a post hoc least significant difference (LSD) or Tamhane test. Data conformed to a normal distribution and homogeneity of variance or were otherwise corrected by SQuare RooT (SQRT) or LOG. Pearson’s correlation analysis was used to determine the correlation between the two results. A significant difference was defined as *p* < 0.05.

## Results

### Voluntary wheel running ameliorated cognitive impairment in APP/PS1 mice

**Fig. 1:**
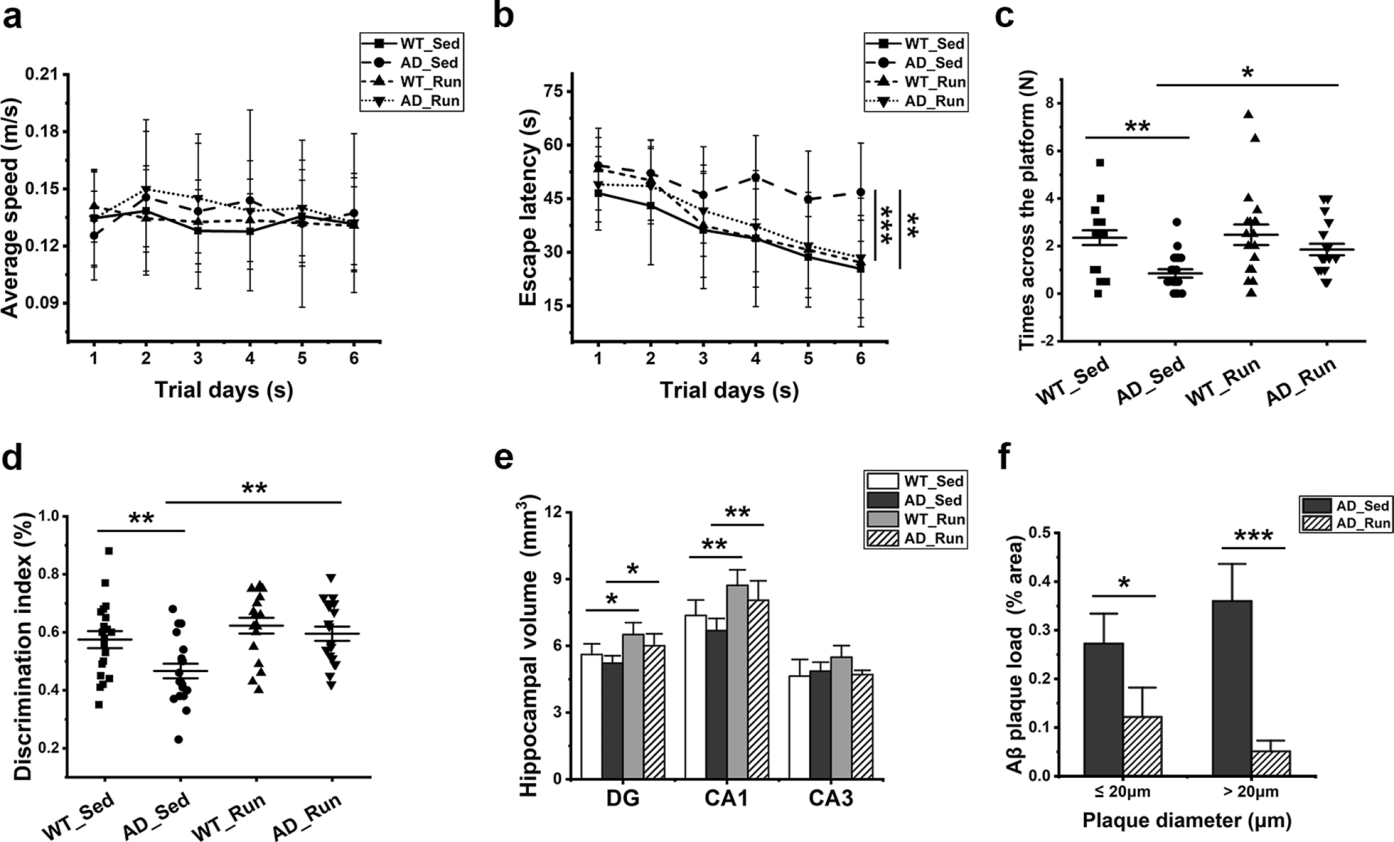
Running exercise prevented cognitive decline and hippocampal atrophy and reduced Aβ plaques in APP/PS1 mice. The effects of long-term voluntary running exercise on the cognitive function of APP/PS1 mice were evaluated by NOR and MWM in 13-month-old WT_Sed, AD_Sed, WT_Run, and AD_Run mice. **a** The average speed in the orientation navigation test. **b** The escape latency in orientation navigation test. **c** The number of times across the platform in the spatial probe test. **d** DI of novel object recognition. **a–d** *n* = 20**. e** The volume of the DG, CA1 and CA3 regions of WT_Sed, AD_Sed, WT_Run, and AD_Run mice. *n* = 5. **f** Quantification results of the amyloid plaque load (% area) for AD_Sed mice and AD_Run mice. *n* = 4–5. Data are shown as the mean ± SD, *p < 0.05, **p < 0.01, ***p < 0.001

There was no significant difference in the average running distance per day (*F* = 0.031, *p* = 0.862) and bodyweight of mice (*F* = 0.099, *p* = 0.960) between the two mouse genotypes (Table 1). The MWM and NOR tests were conducted to evaluate cognitive function. In the MWM test, there was no difference in the average swimming speed in the place navigation test from days 1 to 6 among each group (*F* = 0.388, *p* = 0.762, Fig. 1a), but the escape latency was significantly changed among the four groups (*F* = 7.273, *p* = 0.000, Fig. 1b). Compared with AD_Sed mice, the escape latency of WT_Sed mice was significantly shortened (post hoc, *p* = 0.002, Fig. 1b), and the escape latency of AD_Run mice was significantly shortened (post hoc, *p* = 0.000, Fig. 1b). In the spatial probe test on day 7, the number of times crossed the platform also changed significantly among the four groups (F = 5.824,* p* = 0.001, Fig. 1c). The number of times the AD_Sed mice crossed the platform was less than that of the AD_Run mice (post hoc, *p* = 0.024, Fig. 1c), and the number of times the AD_Sed mice crossed the platform was less than that of the WT_Sed mice (post hoc,* p* = 0.001, Fig. 1c). There was a significant difference in the DI of the NOR test among the four groups (*F* = 6.719, *p* = 0.000, Fig. 1d). The DI significantly increased in AD_Run mice compared with AD_Sed mice (post hoc, *p* = 0.001, Fig. 1d), and the DI significantly increased in WT_Sed mice compared with AD_Sed mice (post hoc, *p* = 0.004, Fig. 1d). These findings demonstrated that long-term voluntary running markedly protected learning and memory loss in 13-month-old APP/PS1 mice.

**Table 1:** Average running distance per day and bodyweight of mice

Group	*N*	Weight (g)	Running distance (km/d)
WT_Sed	20	36.3 ± 4.7	–
AD_Sed	20	35.9 ± 5.0	–
WT_Run	20	35.5 ± 3.9	4.1 ± 1.9
AD_Run	20	36.0 ± 4.2	3.8 ± 2.1

Data are shown as the mean ± SD

### Voluntary running exercise increased hippocampal volume and reduced amyloid plaque deposition in APP/PS1 mice

There were significant differences in the volumes of DG (*F* = 6.490, *p* = 0.004) and CA1 (*F* = 7.558, *p* = 0.002) regions among the four groups, while there was no significant difference in the volume of CA3 region (*F* = 2.909, *p* = 0.067) (Fig. 1e). The volumes of the DG and CA1 regions were significantly increased in AD_Run mice compared with AD_Sed mice (post hoc, *p*(DG) = 0.021, *p*(CA1) = 0.008, Fig. 1e). The volumes of the DG and CA1 regions were significantly increased in WT_Run mice compared with WT_Sed mice (post hoc, *p*(DG) = 0.010, *p*(CA1) = 0.008, Fig. 1e). However, there was no significant difference in the DG, CA1, and CA3 volumes between WT_Sed mice and AD_Sed mice (Fig. 1e). These results indicated that running exercise increased the hippocampal volume in the DG and CA1 regions of APP/PS1 mice relative to their sedentary littermates, as it did in wild-type mice. The Aβ plaque-positive area in the hippocampus of the AD_Run mice was notably lower than that in the hippocampus of AD_Sed mice (*p* = 0.013 (≤ 20 μm in diameter), *p* = 0.000 (> 20 μm in diameter), Fig. 1f), suggesting that running exercise significantly reduced Aβ plaque deposition in the hippocampus of APP/PS1 mice.

### Running exercise led to few DEGs in the hippocampus of APP/PS1 mice

**Fig. 2:**
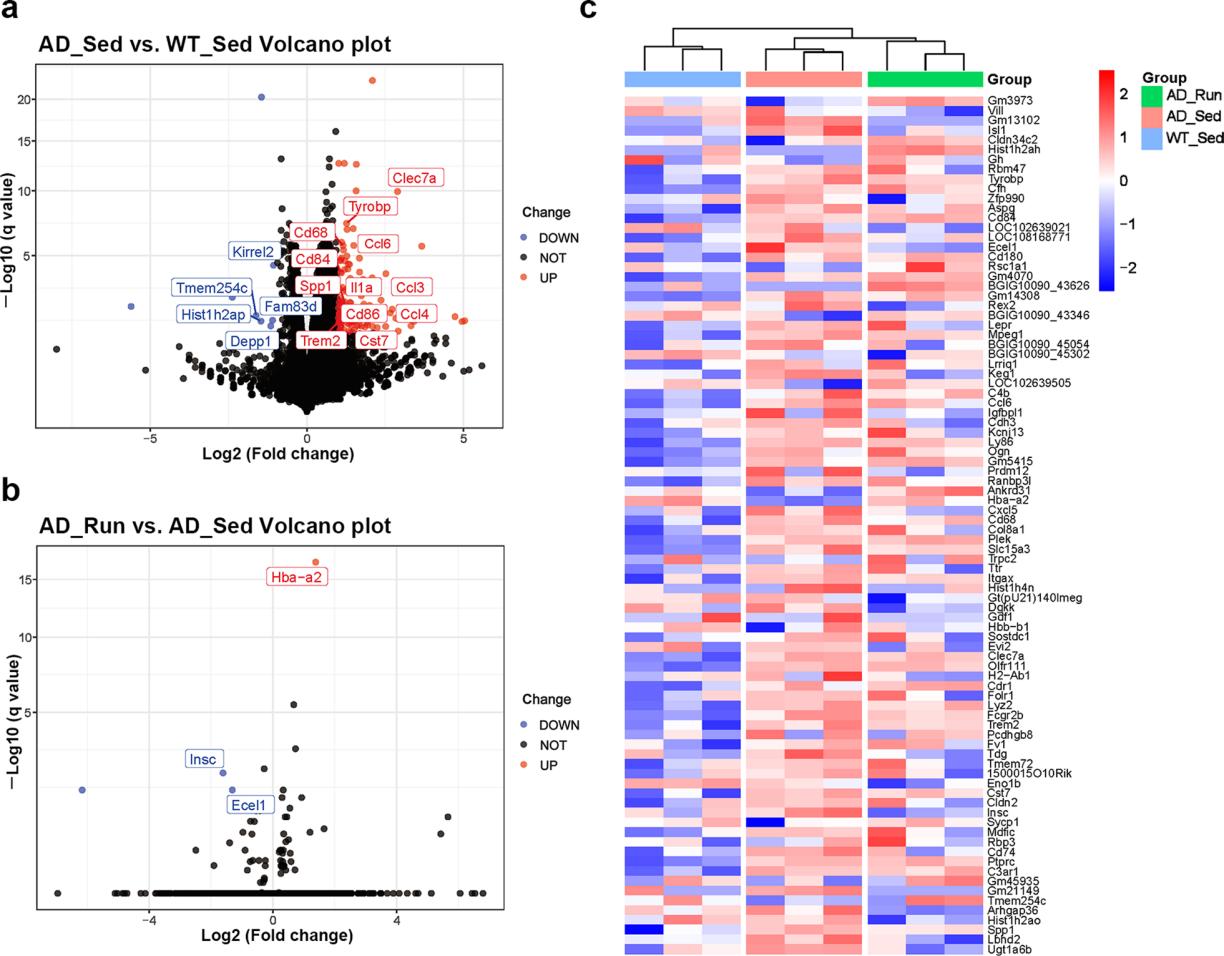
Running exercise led to few DEGs in the hippocampus of APP/PS1 mice. **a** Volcano plot showing the DEGs in AD_Sed mice vs. WT_Sed mice. **b** Volcano plot showing the DEGs in AD_Run mice vs. AD_Sed mice. **c** Heatmap showing the top DEGs in WT_Sed, AD_Sed, and AD_Run mice. *n* = 3. Data are shown as |Log2 (fold change)|≥ 1, adjusted to *p* < 0.05

As shown in Fig. 2a, 115 genes were upregulated and 10 genes were downregulated in AD_Sed mice compared with WT_Sed mice. These upregulated genes included some disease-associated microglial genes, such as Trem2, Tyrobp, Spp1, Clec7a, Ccl3, Cd74, and Cd68, suggesting extensive microglial activation during AD progression. However, there were few DEGs in AD_Run mice compared with AD_Sed mice (Fig. 2b). The top differentially expressed genes are shown in the heatmap (Fig. 2c). These results indicated that the effect of running exercise on the transcriptional level of hippocampal genes was too small to determine molecular mechanisms in APP/PS1 mice, inferring that regulation caused by running exercise may occur at the posttranscriptional level.

### Running exercise enriched in glycometabolic pathways in the hippocampus of APP/PS1 mice

**Fig. 3:**
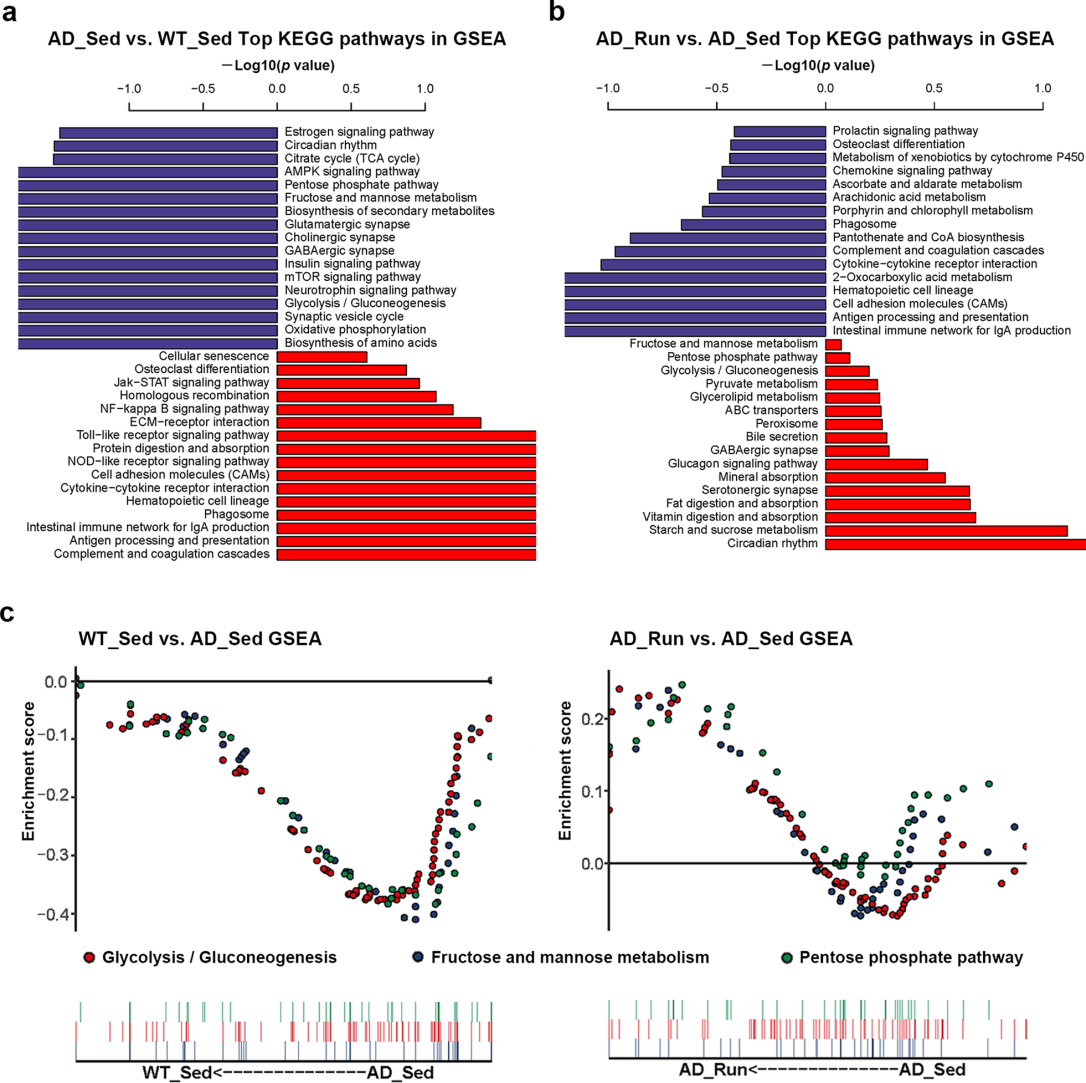
Top KEGG enrichment pathways in GSEA of WT_Sed, AD_Sed and AD_Run mice. **a** Top KEGG enrichment pathways are shown in GSEA of AD_Sed mice vs. WT_Sed mice. **b** Top KEGG enrichment pathways are shown in GSEA of AD_Run mice vs. AD_Sed mice. **c** Several glycometabolic pathways are shown in GSEA of AD_Sed mice vs. WT_Sed mice and AD_Run mice vs. AD_Sed mice. Data are shown as − log[10] (*p* value)

To further observe the slight changes in gene expression, gene set enrichment analysis (GSEA) was performed. The top differential KEGG pathways in GSEA are shown in Fig. 3a, b. We found that several glucose metabolism pathways were reduced in AD_Sed mice compared with WT_Sed mice, including the “pentose phosphate pathway”, “fructose and mannose metabolism” and “glycolysis/gluconeogenesis” (Fig. 3a, c). In contrast, “starch and sucrose metabolism”, “pyruvate metabolism”, “glycolysis/gluconeogenesis”, “pentose phosphate pathway”, and “fructose and mannose metabolism” were upregulated in AD_Run mice compared with AD_Sed mice (Fig. 3b, c). These results suggested that hypometabolism may exist in the hippocampus of APP/PS1 mice, whereas running exercise promoted hippocampal glucose metabolism in the hippocampus of APP/PS1 mice. In addition, several synaptic pathways were reduced in AD_Sed mice compared with WT_Sed mice but upregulated in AD_Run mice compared with AD_Sed mice, such as the “GABAergic synapse” (Fig. 3a, b). PSD95, a postsynaptic density marker, was stained and the mean intensity of PSD95 showed significant difference in the DG (*F* = 9.167, *p* = 0.012), CA1 (*F* = 7.405, *p* = 0.019), and CA3 (*F* = 8.179, *p* = 0.015) regions among the four groups (Additional file 2: Figs. S2a, 2b). The mean intensity of PSD95 in the DG, CA1, and CA3 regions of AD_Sed mice was significantly lower than that in WT_Sed mice (post hoc, *p*(DG) = 0.005, *p*(CA1) = 0.020, *p*(CA3) = 0.005, Additional file 2: Fig. S2b), and the mean intensity of PSD95 in the DG and CA3 regions of AD_Sed mice was also lower than that in AD_Run mice (post hoc, *p*(DG) = 0.008, *p*(CA3) = 0.009, Additional file 2: Fig. S2b). These results suggested that running exercise may improve cognitive function by reducing synapse loss in the hippocampus of APP/PS1 mice.

### Running exercise enhanced hippocampal glucose metabolism in APP/PS1 mice

**Fig. 4:**
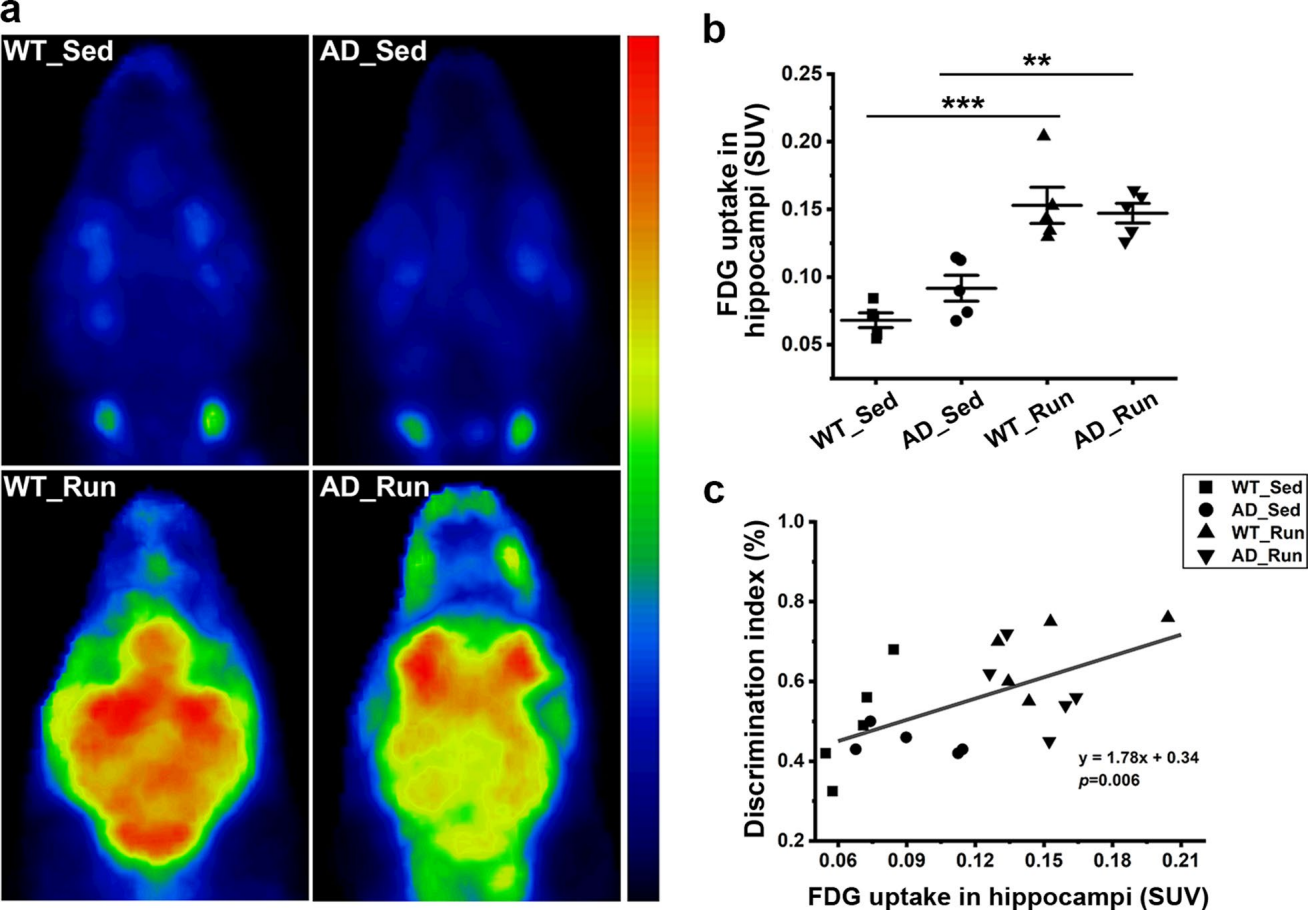
Running exercise enhanced hippocampal glucose metabolism in APP/PS1 mice. **a** Representative [18]F-FDG-μPET 3D reconstruction images of WT_Sed, AD_Sed, WT_Run, and AD_Run mice. **b** Quantification results of the standard uptake value (SUV) in the hippocampus of WT_Sed, AD_Sed, WT_Run, and AD_Run mice. *n* = 5. **c** Pearson’s correlation between the DI of NOR and FDG uptake. Data are shown as the mean ± SD, *p < 0.05, **p < 0.01, ***p < 0.001

To evaluate glucose metabolism in the hippocampus, [18]F-FDG-PET was performed. Our results showed a significant difference in the FDG uptake of the hippocampus among the four groups (*F* = 19.647, *p* = 0.000, Fig. 4a, b). The FDG uptake (SUV) was significantly enhanced in AD_Run mice compared with AD_Sed mice (post hoc, *p* = 0.001, Fig. 4b), confirming the significant enhancement of glucose metabolism in the hippocampus of APP/PS1 mice induced by running exercise. The FDG uptake (SUV) in WT_Run mice was also much higher than that in WT_Sed mice (post hoc, *p* = 0.000, Fig. 4b), indicating that running exercise also increased hippocampal glucose metabolism in WT mice. In addition, hippocampal glucose uptake was significantly correlated with the DI from the NOR test (*p* = 0.006, Fig. 4c).

### Running exercise upregulated microglial GLUT5 but not neuronal GLUT3 in the hippocampus of APP/PS1 mice

**Fig. 5:**
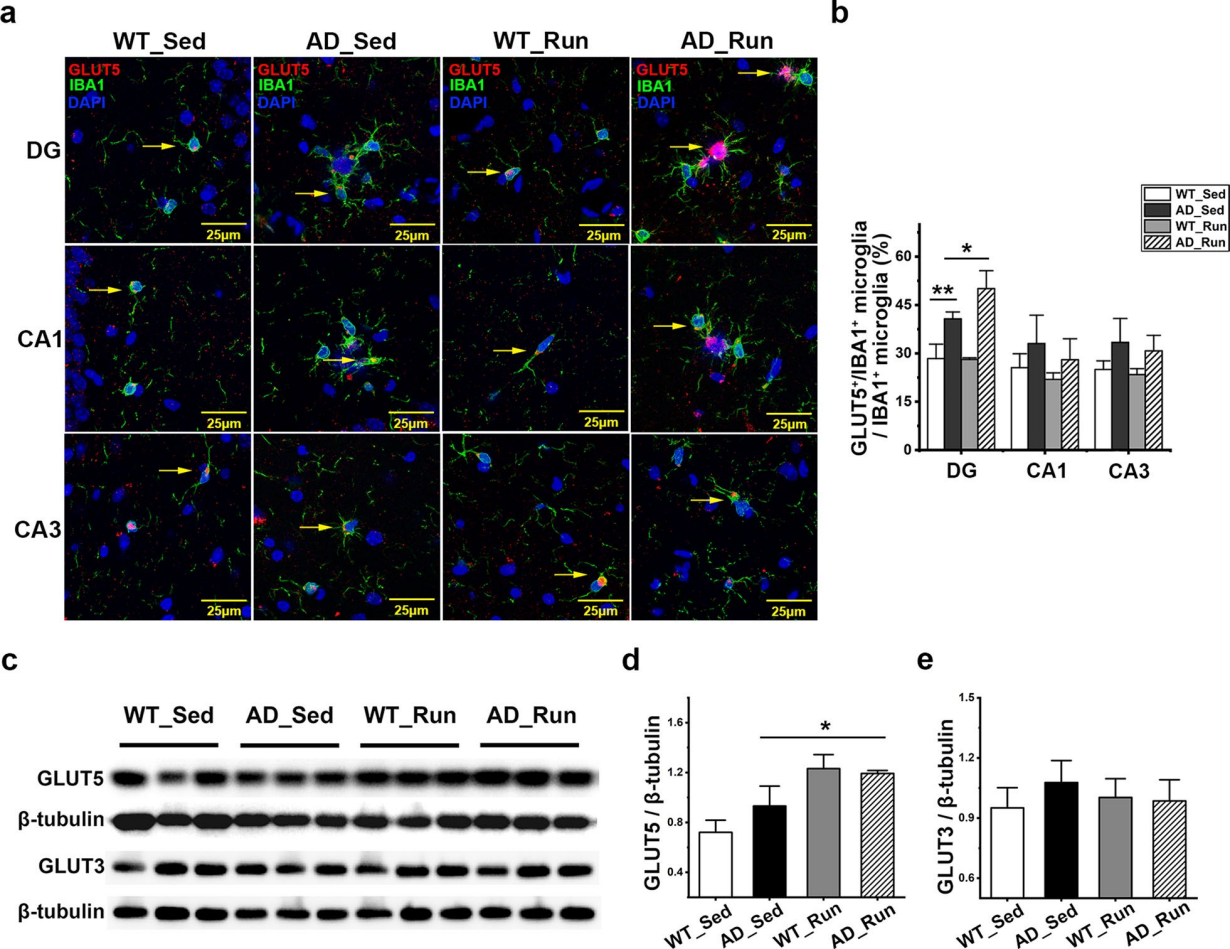
Running exercise upregulated microglial GLUT5 but not neuronal GLUT3 in APP/PS1 mice. **a** Representative immunofluorescence staining of GLUT5 (red), IBA1 (green), and DAPI (blue) in the DG, CA1 and CA3 regions of WT_Sed, AD_Sed, WT_Run, and AD_Run mice. Scale bar: 25 μm. **b** Quantification results of the ratio of GLUT5^+^/IBA1^+^ microglia in the hippocampus of WT_Sed, AD_Sed, WT_Run, and AD_Run mice. **c** GLUT5 and GLUT3 expression in the hippocampus of WT_Sed, AD_Sed, WT_Run, and AD_Run mice is shown by western blotting. **d, e** Quantification results of GLUT5/β-tubulin and GLUT3/β-tubulin. n = 3. Data are shown as the mean ± SD, *p < 0.05, **p < 0.01, ***p < 0.001

As shown in Fig. 5a, GLUT5 was mainly expressed on microglia. There was a significant difference in the ratio of GLUT5^+^/IBA1^+^ microglia in the DG region (*F* = 25.520, *p* = 0.000), but not in the CA1 (*F* = 1.830, *p* = 0.220) or CA3 (*F* = 3.026, *p* = 0.094) regions among the four groups (Fig. 5a, b). The ratio of GLUT5^+^/IBA1^+^ microglia in the DG was significantly increased in AD_Sed mice compared with WT_Sed mice (post hoc, *p* = 0.002, Fig. 5b). Furthermore, the ratio of GLUT5^+^/IBA1^+^ microglia in the DG was significantly increased in AD_Run mice compared with AD_Sed mice (post hoc, *p* = 0.021, Fig. 5b). The protein level of GLUT5 was also changed significantly in the hippocampus among the four groups (*F* = 7.087, *p* = 0.012, Fig. 5c, d). Although the expression of GLUT5 was not significantly different between WT_Sed mice and AD_Sed mice (post hoc, *p* = 0.189, Fig. 5c, d), the expression of GLUT5 was significantly increased in AD_Run mice compared with AD_Sed mice (post hoc, *p* = 0.017, Fig. 5c, d). However, there was no significant difference in the GLUT3 expression among the four groups (*F* = 1.240, *p* = 0.357, Fig. 5c, e). These results indicated that running exercise may improve cognition through enhancing microglial glucose metabolism in APP/PS1 mice.

### Running exercise maintained hippocampal TREM2 protein levels and reduced plasma sTREM2 levels in APP/PS1 mice

**Fig. 6:**
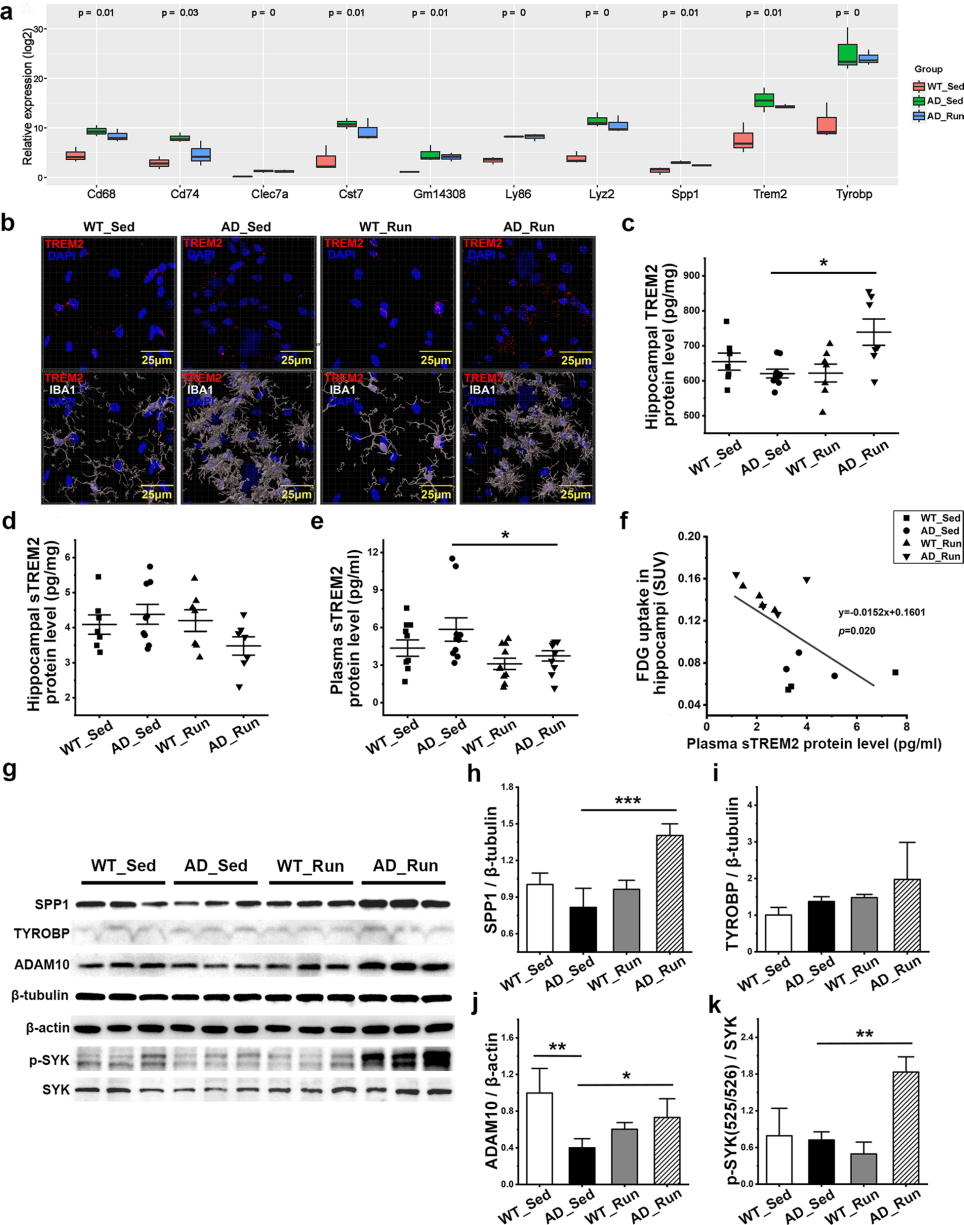
Running exercise maintained TREM2 levels and reduced sTREM2 levels in APP/PS1 mice. **a** Relative expression of several top DEGs among WT_Sed mice, AD_Sed mice, and AD_Run mice. **b** Representative z-stack images (0.22 μm steps) of TREM2 (red) and IBA1 (white) microglia in the DG of WT_Sed, AD_Sed, WT_Run, and AD_Run mice and TREM2-positive puncta outside IBA1 were masked. Scale bar: 25 μm. **c, d** Levels of TREM2 and sTREM2 in the hippocampus of WT_Sed, AD_Sed, WT_Run, and AD_Run mice were determined by ELISA. *n* = 7–9. **e** The level of sTREM2 in the plasma of WT_Sed, AD_Sed, WT_Run, and AD_Run mice was determined by ELISA. *n* = 10. **f** Pearson’s correlation between plasma sTREM2 levels and hippocampal FDG uptake. **g** The expression of SPP1, TYROBP, ADAM10, p-SYK (Tyr525/526), and SYK in the hippocampus of WT_Sed, AD_Sed, WT_Run, and AD_Run mice was detected by western blotting. **h–k** Quantification results of SPP1/β-tubulin, TYROBP/β-tubulin, ADAM10/β-actin, and p-SYK/SYK. *n* = 3. Data are shown as the mean ± SD, *p < 0.05, **p < 0.01, ***p < 0.001

As shown in Fig. 6a, running exercise did not affect several microglial genes at the mRNA level that were upregulated in APP/PS1 mice, such as Trem2, Tyrobp, and Spp1. The mRNA levels of Trem2, Tyrobp, Cd68, Cd74, Itgax, and Spp1 in the hippocampus were also verified using qRT–PCR. There were significant differences in the mRNA levels of Trem2 (*F* = 9.257, *p* = 0.001), Tyrobp (*F* = 9.030, *p* = 0.001), Cd68 (*F* = 8.145, *p* = 0.002), Cd74 (*F* = 19.357, *p* = 0.000), and Itgax (*F* = 39.541, *p* = 0.000) among the four groups, while there was no significant difference in the mRNA levels of Spp1 among the four groups (*F* = 1.463, *p* = 0.252) (Additional file 3: Fig. S3a–f). The mRNA levels of Trem2, Tyrobp, Cd68, Cd74, and Itgax was significantly increased in AD_Sed mice compared with WT_Sed mice (post hoc, *p* = 0.000, *p* = 0.017, *p* = 0.006, *p* = 0.000, *p* = 0.000). However, there were no significant differences in the level of these genes between AD_Run mice and AD_Sed mice except for a decrease in Cd74 in the hippocampus of AD_Run mice compared with AD_Sed mice (post hoc, *p* = 0.001, Additional file 3: Fig. S3d). Therefore, we evaluated the protein levels of TREM2 and sTREM2. There were significant differences in the protein levels of TREM2 in the hippocampus (*F* = 4.440, *p* = 0.012, Fig. 6c) and the level of sTREM2 in the plasma (*F* = 3.307, *p* = 0.031, Fig. 6e), but there was no significant difference in the level of sTREM2 in hippocampus among groups (*F* = 1.856, *p* = 0.162, Fig. 6d). We found more TREM2-positive fluorescent labels in the microglia of AD_Run mice (Fig. 6b). The levels of TREM2 in the hippocampus were significantly increased in AD_Run mice compared with AD_Sed mice (post hoc, *p* = 0.003, Fig. 6c). These results indicated that running exercise upregulated the expression of the TREM2 protein in the hippocampus of APP/PS1 mice. However, the levels of sTREM2 were significantly reduced in AD_Run mice compared with AD_Sed mice in the plasma (post hoc, *p* = 0.027, Fig. 6e), suggesting that running exercise may reduce TREM2 shedding and prevent sTREM2 release into the blood in APP/PS1 mice. Moreover, there was a significant negative correlation between plasma sTREM2 levels and hippocampal FDG uptake (*p* = 0.020, Fig. 6f), which indicated that TREM2 hydrolysis was negatively associated with hippocampal glucose metabolism. Inhibiting the loss of TREM2 may be one of the mechanisms by which running exercise improved glucose metabolism in the hippocampus and rescued cognitive decline in APP/PS1 mice.

The protein level of SPP1, a TREM2-dependent protein [33] (*F* = 18.293, *p* = 0.001, Fig. 6h), ADAM10, a protease of TREM2 (*F* = 6.679, *p* = 0.014, Fig. 6j), and p-SYK (*F* = 12.638, *p* = 0.002, Fig. 6k) were significantly different among groups, but TYROBP (DAP12) expression was unchanged significantly (*F* = 2.108, *p* = 0.178, Fig. 6i). SPP1 was significantly increased in AD_Run mice compared with AD_Sed mice (post hoc, *p* = 0.000, Fig. 6g, h). ADAM10 showed lower expression levels in AD_Sed mice than in WT_Sed mice (post hoc, *p* = 0.002, Fig. 6g, j) and AD_Run mice (post hoc, *p* = 0.042, Fig. 6g, j), which suggested that there might be other reasons for the increased hydrolysis of TREM2 in APP/PS1 mice. The ratio of p-SYK (Tyr525/526)/SYK increased significantly in AD_Run mice compared with AD_Sed mice (post hoc, *p* = 0.002, Fig. 6g, k,), suggesting that running exercise enhanced the phagocytosis of microglia by activating the TREM2/SYK signaling pathway.

### Running exercise increased the total number and branches of microglia in the DG of APP/PS1 mice

**Fig. 7:**
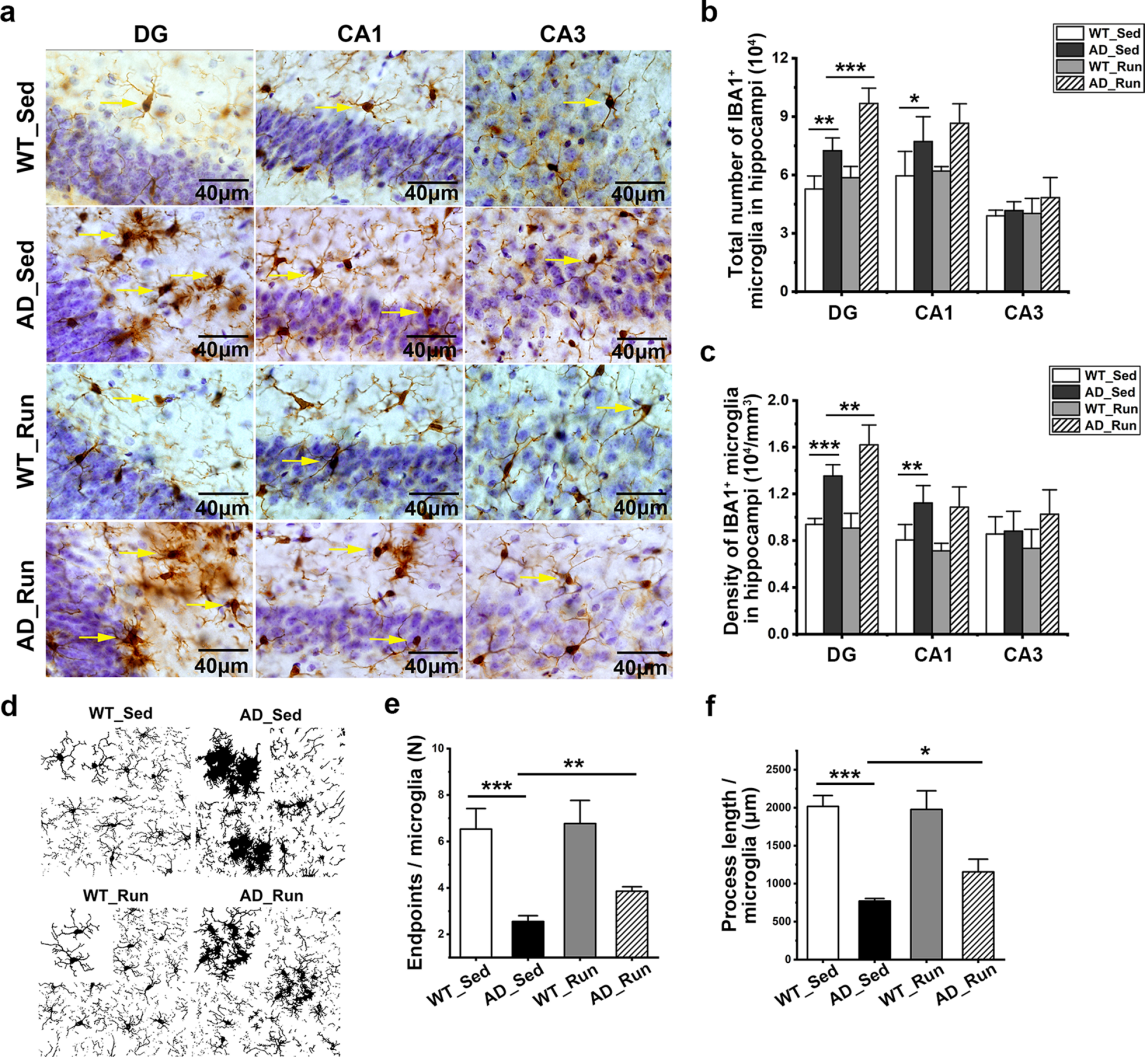
Running exercise increased the total number of microglia in the DG of APP/PS1 mice. **a** Representative immunohistochemical staining of IBA1-labeled microglia in the DG, CA1, and CA3 regions of WT_Sed, AD_Sed, WT_Run, and AD_Run mice. Scale bar: 40 μm. **b, c** Quantification results of the total number and density of IBA1^+^ microglia in the DG, CA1, and CA3 regions of WT_Sed, AD_Sed, WT_Run, and AD_Run mice. **d** Representative binary images showing IBA1^+^ microglia with the uniform protocol in the DG of WT_Sed, AD_Sed, WT_Run, and AD_Run mice. **e, f** Quantification results of endpoints/microglia and process length/microglia. *n* = 4–5. Data are shown as the mean ± SD, *p < 0.05, **p < 0.01, ***p < 0.001

The total number of microglia in the DG (*F* = 41.644, *p* = 0.000) and CA1 (*F* = 7.907, *p* = 0.002) were significantly different among the four groups (Fig. 7a, b). The total number of microglia in the DG and CA1 increased significantly in AD_Sed mice compared with WT_Sed mice (post hoc, *p*(DG) = 0.001, *p*(CA1) = 0.020, Fig. 7a, b), suggesting significant microglial proliferation in the hippocampus of APP/PS1 mice. Furthermore, the total number of microglia in the DG was significantly increased in AD_Run mice compared with AD_Sed mice (post hoc, *p* = 0.000, Fig. 7a, b). There was no significant difference in the total number of microglia in the CA1 region between AD_Sed mice and AD_Run mice (post hoc, *p* = 0.188, Fig. 7a, b). There was no significant difference in the total number of microglia in the CA3 region among the four groups of mice (*F* = 1.738, *p* = 0.202, Fig. 7a, b). These results indicated that running exercise led to an increase in microglia in the DG of APP/PS1 mice. There was also a significant difference in the density of IBA1 microglia in the DG region among the four groups (*F* = 39.990, *p* = 0.000, Fig. 7c). The density of IBA1 microglia in the DG of AD_Run mice was higher than that in the DG of AD_Sed mice (post hoc, *p* = 0.005, Fig. 7c). The endpoints of microglial branches (*F* = 28.368, *p* = 0.000) and the process length of microglia (*F* = 46.012, *p* = 0.000) were changed significantly in the DG region among the four groups (Fig. 7d–f). The endpoints of microglial branches were reduced in the DG of AD_Sed mice compared with WT_Sed mice (post hoc, *p* = 0.000, Fig. 7d, e). In addition, the endpoints of microglial branches were increased in the DG of AD_Run mice compared with AD_Sed mice (post hoc, *p* = 0.003, Fig. 7d, e). Similarly, the process length of microglia decreased in the DG of AD_Sed mice compared with WT_Sed mice (post hoc, *p* = 0.000, Fig. 7d, f), and the process length of the microglia increased in the DG of AD_Run mice compared with AD_Sed mice (post hoc, *p* = 0.044, Fig. 7d, f). Collectively, our results showed that running exercise increased the number of microglia and improved the number and length of microglial branches in the DG of APP/PS1 mice.

### Running exercise did not promote the proliferation of microglia in the hippocampus of APP/PS1 mice

**Fig. 8:**
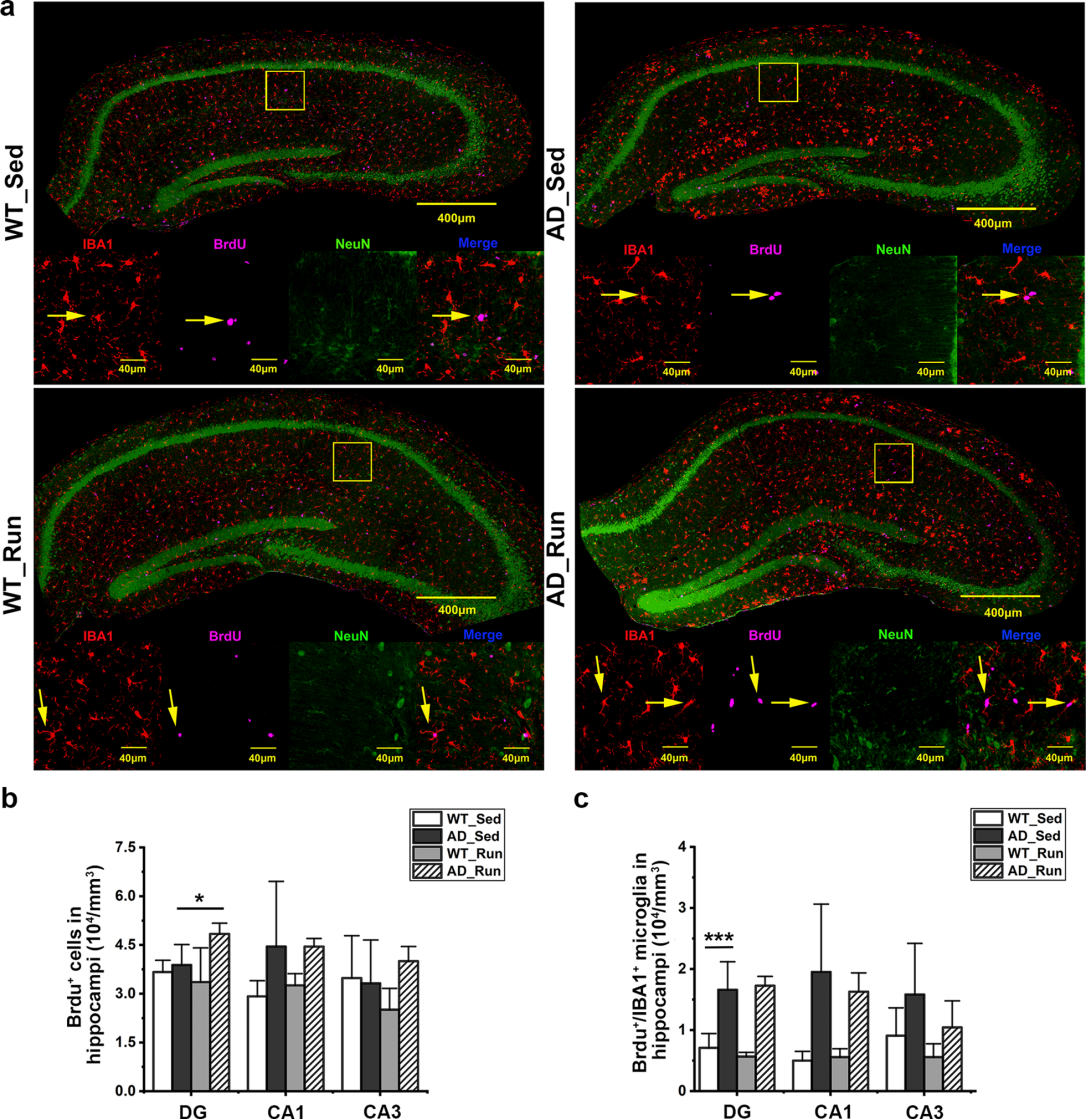
Running exercise did not promote microglial proliferation in the hippocampus of APP/PS1 mice. **a** Representative immunofluorescence staining of BrdU (purple), IBA1 (red), and NeuN (green) in the hippocampus of WT_Sed, AD_Sed, WT_Run, and AD_Run mice. Scale bar: 40 μm. **b** Quantification results of the number of BrdU^+^ cells in the DG, CA1, and CA3 regions of WT_Sed, AD_Sed, WT_Run, and AD_Run mice. **c** Quantification results of the number of BrdU^+^/IBA1^+^ microglia in the DG, CA1, and CA3 regions of WT_Sed, AD_Sed, WT_Run, and AD_Run mice. n = 4–5. Data are shown as the mean ± SD, *p < 0.05, **p < 0.01, ***p < 0.001

To further determine whether the effect of running exercise on the number of microglia is due to prompted proliferation or survival, microglia were labeled with BrdU, a marker of proliferation [42], and NeuN was used to delineate hippocampal subregions. There was a significant difference in the number of BrdU^+^/IBA1^+^ microglia in the DG region among the four groups (*F* = 22.959, *p* = 0.000, Fig. 8a, c). The number of BrdU^+^/IBA1^+^ microglia in the DG was significantly increased in AD_Sed mice compared with WT_Sed mice (post hoc, *p* = 0.000, Fig. 8a, c), suggesting significant microglial proliferation in the hippocampus of APP/PS1 mice. Although the number of BrdU^+^ cells in the DG of the hippocampus was increased in AD_Run mice compared with AD_Sed mice (post hoc, *p* = 0.044, Fig. 8a, b), there was no difference in the number of BrdU^+^/IBA1^+^ microglia in the hippocampus between AD_Run mice and AD_Sed mice (post hoc, *p* = 0.709, Fig. 8c). These results suggested that there was significant microglial proliferation in the DG of APP/PS1 mice and that running exercise did not further promote microglial proliferation. The increase in microglial number in the DG may be due to the promotion of microglial survival.

### Running exercise reduced the immunoactivity of CD68 in the hippocampus of APP/PS1 mice

**Fig. 9:**
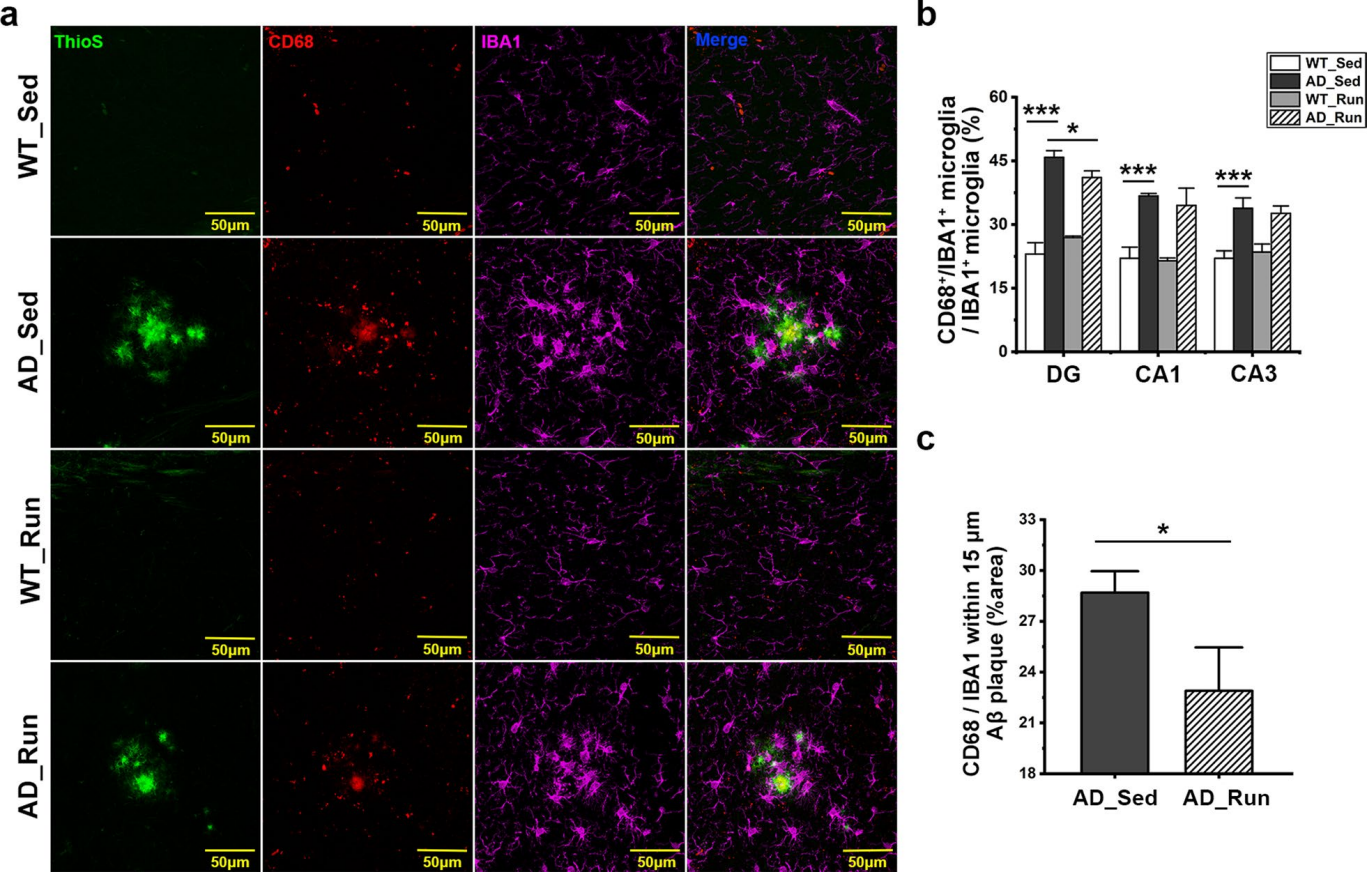
Running exercise reduced the immunoactivity of CD68 in the hippocampus of APP/PS1 mice. **a** Representative immunofluorescence staining of ThioS (green), CD68 (red), and IBA1 (purple) in the hippocampus of WT_Sed, AD_Sed, WT_Run, and AD_Run mice. Scale bar: 50 μm. **b** Quantification results of the ratio of CD68^+^/IBA1^+^ microglia in the DG, CA1, and CA3 regions of WT_Sed, AD_Sed, WT_Run, and AD_Run mice. **c** Quantification results of the ratio of the CD68/IBA1-positive area around Aβ plaques (≤ 15 μm) in the hippocampus of AD_Sed and AD_Run mice. *n* = 3. Data are shown as the mean ± SD, *p < 0.05, **p < 0.01, ***p < 0.001

CD68, a marker of microglial activation, and IBA1 were stained. There were significant differences in the ratio of CD68^+^/IBA1^+^ microglia in the DG (*F* = 116.905, *p* = 0.000), CA1 (*F* = 32.310, *p* = 0.000), and CA3 (*F* = 28.301, *p* = 0.000) regions among the four groups (Fig. 9a, b). The ratio of CD68^+^/IBA1^+^ microglia in the DG, CA1, and CA3 regions was significantly increased in AD_Sed mice compared with WT_Sed mice (post hoc, *p*(DG) = 0.000, *p*(CA1) = 0.000, *p*(CA3) = 0.000, Fig. 9b), while the ratio of CD68^+^/IBA1^+^ microglia in the DG region of AD_Run mice was lower than that of AD_Sed mice (post hoc, *p* = 0.011, Fig. 9b). Moreover, the ratio of the CD68/IBA1-positive area around Aβ plaques (≤ 15 μm) in the hippocampus of AD_Run mice was significantly lower than that in AD_Sed mice (*p* = 0.025, Fig. 9a, c). These results suggested that running exercise might reduce the proportion of microglial activation in APP/PS1 mice.

## Discussion

The benefits of physical exercise on cognitive function have been widely recognized. Voluntary wheel running by mice is more akin to physical exercise in humans [54], because voluntary running exercise follows the circadian rhythms of mice and does not cause a substantial amount of stress [55]. Therefore, we used a voluntary running intervention to study the underlying mechanisms by which running ameliorates AD. Studies have reported that voluntary running exercise promotes cognitive function in AD model mice [56, 57]. However, in these studies, 3–5 mice were housed together and shared one running wheel. In our study, the running distance of each mouse was evaluated, and there was no significant difference in running distance between WT mice and AD model mice during the intervention. Our study excluded experimental errors due to exercise intensity. Consistent with most articles [56, 58], our results showed that long-term voluntary running significantly alleviated cognitive impairment, increased hippocampal volume in the DG and CA1 regions, reduced hippocampal Aβ deposition, and prevented synaptic loss in APP/PS1 mice.

Emerging evidence has identified several targets for exercise to improve cognition in AD model mice [44, 59]. However, few mechanisms have been reported macroscopically. In our study, GSEA showed that glucose metabolism-associated pathways were significantly downregulated in AD_Sed mice compared with WT_Sed mice, but upregulated in AD_Run mice compared with AD_Sed mice. These results suggested that there may be a decrease in glucose metabolism during AD and that running exercise may promote cognitive function by regulating hippocampal glucose metabolism. In line with our results, decreased brain glucose metabolism has also been established using [18]F-FDG-PET in both patients with AD and AD model mice [11–13]. However, there are still some conflicting results regarding FDG uptake in AD model mice [60]. Increased FDG uptake was reported in the hippocampus of APP/PS1 mice at the age of 5 months [61] and 12 months [62]. Our previous study also found that hippocampal FDG uptake increased in 10-month-old APP/PS1 mice (Additional file 1: Fig. S1b, c). We speculated that these discrepancies may be related to the pathologic processes of AD. For example, early Aβ deposition induced hypermetabolism in APP/PS1 mice [63], and higher [18]F-florbetapir uptake was observed in preclinical AD patients [64]. To the best of our knowledge, only one study has reported that 6 months of aerobic training increases hippocampal FDG uptake in patients with MCI [18]. However, no study has investigated the effects of long-term running exercise on hippocampal glucose metabolism in AD. Our results showed that running exercise significantly increased hippocampal FDG uptake in APP/PS1 mice, providing evidence that long-term voluntary running exercise promotes hippocampal glucose metabolism in APP/PS1 mice.

Glucose transporters are important for glucose uptake in the brain and classical indicators of this uptake [65]. GLUT1 is expressed in oligodendrocytes and astrocytes [66], GLUT3 is expressed in neurons, and GLUT5 is expressed in microglia [67]. Reduced GLUT1 and GLUT3 have been reported in AD [68, 69]. However, there have been no studies on the changes in GLUT5 in AD. In our study, we found that although there was no significant difference in GLUT5 expression between APP/PS1 mice and WT mice, the ratio of GLUT5-positive microglia was increased in the DG of APP/PS1 mice, in parallel with the microglial increase, suggesting increased glucose demand of microglia in AD pathology. It has been reported that treadmill exercise and regular swimming exercise increase the protein expression of GLUT1 and GLUT3 in the AD mouse brain [70, 71]. Our results showed that voluntary running exercise increased both GLUT5 expression and the ratio of GLUT5-positive microglia in APP/PS1 mice, suggesting that increased microglial glucose metabolism is as important as increased GLUT1 and GLUT3 in exercise benefits in AD. Promoting microglial glucose metabolism may be one of the mechanisms by which running exercise delays AD progression.

What might be the underlying mechanism by which running exercise improves microglial glucose metabolism? TREM2 has been shown to play an important role in microglial metabolic activity [32, 33]. Nevertheless, studies have reported that the changes in TREM2 expression at the protein level were not parallel to the gene expression level in AD. Roussos et al. found that Trem2 and Tyrobp gene expression was upregulated, whereas TREM2 protein expression was downregulated and TYROBP protein expression was not significantly changed in the superior temporal gyrus of AD patients [72]. Our results showed that the gene expression levels of Trem2, Tyrobp, and Spp1 were significantly upregulated in the hippocampus of APP/PS1 mice; however, the protein levels of these genes did not change significantly in the hippocampus of APP/PS1 mice compared with WT mice. These inconsistent results may be caused by progressive pathological toxicity in AD. Previous studies have reported that the protein expression of TREM2 was significantly decreased in Aβ-injected mice [73] or lipopolysaccharide-stimulated APP/PS1 mice [74], suggesting that the level of the TREM2 protein gradually decreases with continuous neuroinflammatory toxicity in AD. On the other hand, we also demonstrated that running exercise significantly upregulated the protein levels of TREM2 and SPP1 in the hippocampus of APP/PS1 mice. Baik et al. showed that treatment with recombinant interferon-γ (IFN-γ) reversed metabolic defects in microglia and alleviated AD pathology in 5xFAD mice [75] and that TREM2 could be activated by IFN-γ [76]. Our results, together with previous findings, suggested that running exercise-induced TREM2 protein upregulation may be associated with the promotion of microglial glucose metabolism and cognitive function in APP/PS1 mice.

How does running exercise increase TREM2 protein levels in AD? Many studies have confirmed that the level of sTREM2 in the CSF of AD patients is significantly increased [37–39], but there was no difference in plasma sTREM2 levels between healthy controls and patients with MCI or AD [77]. In our study, we showed that the levels of sTREM2 in the hippocampus and plasma did not change significantly between APP/PS1 mice and WT mice. However, the levels of sTREM2 in the plasma decreased significantly in APP/PS1 running mice compared with APP/PS1 mice. Our results suggested that running exercise may inhibit the shedding of TREM2 to maintain TREM2 protein levels. Moreover, we also found that there was a negative correlation between the plasma sTREM2 level and hippocampal FDG uptake. Due to the close relationship between TREM2 and brain glucose metabolism [35], our results further suggested that running exercise-induced inhibition of TREM2 hydrolysis may be associated with the promotion of hippocampal glucose metabolism. We also tried to find possible reasons for the increase in sTREM2 levels. We detected ADAM10 [37], a protease of TREM2, but the expression of ADAM10 was increased in APP/PS1 running mice. One study supported our results that treadmill exercise upregulated ADAM10 and promoted nonamyloid production pathways in AD model mice [78]. Feuerbach et al. showed that ADAM17 was the main protease involved in releasing the extracellular domain of TREM2, while ADAM10 plays a secondary role [79]. Therefore, the mechanisms of the downregulation of sTREM2 in the hippocampus and plasma of APP/PS1 running mice need to be further studied.

Given that TREM2 maintains microglial metabolic activity to support microglial survival [33], it is unclear whether the upregulation of TREM2 induced by running exercise is accompanied by a change in the number of microglia. Therefore, we evaluated the total number of microglia using stereological methods. Several semiquantitative studies have reported the effects of long-term running exercise on microglial intensity in AD mice [80, 81]. However, the unbiased estimation of the number of cells of interest by the stereological method is accurate [82]. Using the unbiased stereological method, Hashiguchi et al. reported that 4 weeks of ladder-climbing exercise did not affect the total number of microglia in the dorsal hippocampus of APP/PS1 mice at the age of 8 months [83]; however, they did not quantify the number of microglia in the subregions of the hippocampus. In our study, we showed that long-term voluntary running significantly increased the total number of microglia in the DG of 13-month-old APP/PS1 mice. The difference between our results and Hashiguchi’s results may be due to differences in the exercise methods, age of the mice and hippocampal subregions. Our results indicated that the effect of running exercise on microglia in AD mice has regional characteristics, and long-term voluntary running increased the total number of microglia in the DG of APP/PS1 mice.

Regarding the effect of running exercise on microglial proliferation, it has been reported that voluntary running exercise could increase [84] or decrease [85] the number of BrdU^+^/IBA1^+^ microglia in WT mice. Whether running exercise affects the proliferation of microglia in the hippocampus of APP/PS1 mice has not been reported. We found that running exercise did not change the number of BrdU^+^/IBA1^+^ microglia in the hippocampus of AD mice. Therefore, the increase in microglial number induced by running exercise might not be due to microglial proliferation but to microglial survival. We also examined the microglial morphology. Although Rodriguez et al. reported that 9 months of voluntary running promoted an increase in microglial surface area and microglial body volume in 3xTG-AD mice [86], we used skeleton analysis to show that long-term running significantly increased the length and endpoints of microglial branches in the DG of APP/PS1 mice. Our results further confirmed that long-term running exercise increased the number and length of microglial branches in APP/PS1 mice. Moreover, we found that running exercise reduced the ratio of CD68^+^/IBA1^+^ microglia in the DG of APP/PS1 mice. Although running exercise did not affect CD68 mRNA levels in APP/PS1 mice (Fig. S3c), it is possible that running exercise reduces the ratio of microglial activation and thus subsequent neuroinflammation. This is supported by previous studies showing that running exercise reduced microglial activation and proinflammatory factors in APP/PS1 mice [81, 87]. Together, microglia might be one of the structural targets involved in running exercise mitigating AD cognitive impairment.

## Conclusions

In our study, we found that long-term voluntary running improved cognitive impairment in AD model mice. Our results provide evidence that long-term voluntary running exercise inhibits the shedding of TREM2 and maintains TREM2 protein levels, which are associated with the exercise-induced promotion of brain glucose metabolism and microglial glucose metabolism in the hippocampus of AD mice and the exercise-induced morphological plasticity of their hippocampal microglia. Our results suggested that hippocampal microglia may be a structural target responsible for the benefits of running exercise in AD, and promoting hippocampal glucose metabolism and microglial function and morphology modulated by TREM2 might be a novel strategy for AD treatment.

## Supplementary Information

**Additional file 1: Figure S1. **Hippocampal glucose metabolism increased in 10-month-old APP/PS1 mice. **a** The predefined mouse brain VOI template is shown. RHIP: Right hippocampus. **b** Representative [18]F-FDG-μPET 3D images showing FDG uptake of WT_Sed mice and AD_Sed mice at 10 months of age. **c** Quantification results of the standard uptakevalue (SUV) in the hippocampus between WT_Sed mice and AD_Sed mice at the ages of 10 months and 13 months. n = 10 at 10 months of age, n = 5 at 13 months of age. Paired *t-test* was applied between two groups. Data are shown as the mean ± SD, * *p *<0.05, ** *p *< 0.01, *** *p *< 0.001.

**Additional file 2: Figure S2. **Running exercise enhanced the immunoactivity of PSD95 in the hippocampus of APP/PS1 mice. **a** Representative immunofluorescence staining of PSD95 (red) and DAPI (blue) in the DG, CA1, and CA3 regions of WT_Sed, AD_Sed, WT_Run, and AD_Run mice. Scale bar: 50 μm. **b** Quantification results of the mean intensity of PSD95 in the DG, CA1, and CA3 regions of WT_Sed, AD_Sed, WT_Run, and AD_Run mice. n = 3. Data are shown as the mean ± SD, * *p *< 0.05, ** *p *< 0.01,*** *p *< 0.001.

**Additional file 3: Figure S3.** Relative mRNA levels of microglia-related genes in the hippocampus. **a-f** Quantification results of the relative mRNA levels of Trem2, Tyrobp, Cd68, Cd74, Itgax, and SPP1 in the hippocampus of WT_Sed, AD_Sed, WT_Run, and AD_Run mice. n = 3. Data are shown as the mean ± SD, * *p *<0.05, ** *p *< 0.01, *** *p *< 0.001.

## Data Availability

All data are available from the corresponding author upon reasonable request.

## Abbreviations

AD: Alzheimer’s disease
WT: Wild type
APP: Amyloid precursor protein
Aβ: β-Amyloid
AOPE: Apolipoprotein E
Sed: Sedentary
MCI: Mild cognitive impairment
CSF: Cerebrospinal fluid
NOR: New object recognition
DI: Discrimination index
MWM: Morris water maze
ANOVA: Analysis of variance
RNA-seq: RNA sequencing
DEGs: Differentially expressed genes
KEGG: Kyoto encyclopedia of genes and genomes
GESA: Gene set enrichment analysis
TCA cycle: Citrate cycle
[18]F-FDG-PET: [18]F-fluoro-2-deoxy-d-glucose/positron emission tomography
SUV: Standard uptake value
TSPO: Translocation protein 18 kDa
VOI: Volume of interest
PBS: Phosphate-buffered saline
ELISA: Enzyme-linked immunosorbent assay
DAPI: 4′,6-Diamidino-2-phenylindole
DG: Dentate gyrus
IBA1: Induction of brown adipocytes 1
BrdU: 5-Bromo-2′-deoxyuridine
NeuN: Neuronal nuclear antigen
TREM2: Triggering receptor expressed in myeloid cells 2
sTREM2: Soluble TREM2
TREM2-L: TREM2 ligand
DAP12: DNAX-activation protein 12
TYROBP: TYRO protein tyrosine kinase binding protein
ITAM: Immunoreceptor tyrosine-based activation motif
SPP1: Secreted phosphoprotein 1
SYK: Spleen tyrosine kinase
ADAM: A disintegrin and metallopeptidase
CD68: Clostridioides difficile CD68
CD74: MHC class II invariant chain
ITGAX: Integrin subunit alpha X
AMPK: AMP-activated protein kinase
mTOR: Mammalian target of rapamycin
GLUT: Glucose transporter
IFN-γ: Interferon-γ
PSD95: Postsynaptic density protein 95
