# Supplementary figures and images for: Long-term running exercise improves cognitive function and promotes microglial glucose metabolism and morphological plasticity in the hippocampus of APP/PS1 mice
Source: J Neuroinflammation. 2022 Feb 5;19:34. doi: 10.1186/s12974-022-02401-5 (PMC8817568; doi:10.1186/s12974-022-02401-5)

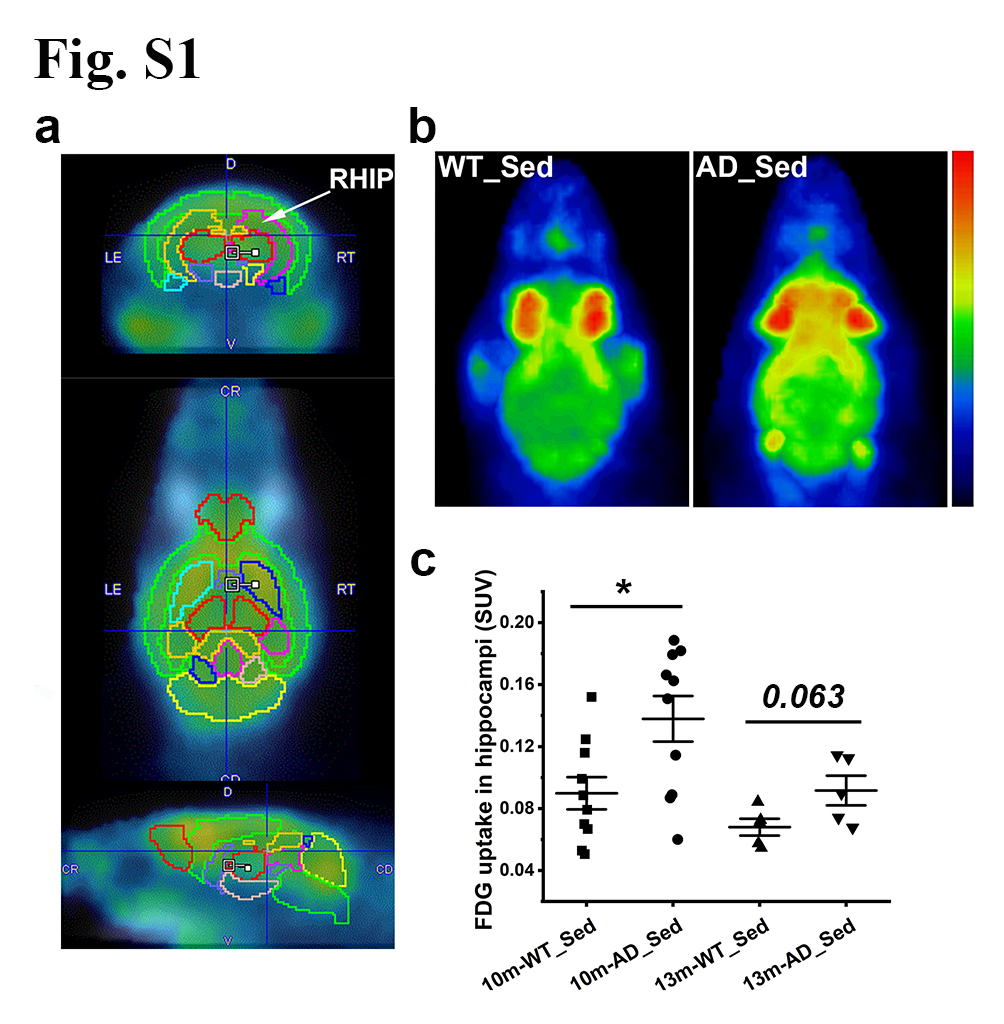

Supplement: Supplementary file 1 — Additional file 1: Figure S1. Hippocampal glucose metabolism increased in 10-month-old APP/PS1 mice. a The predefined mouse brain VOI template is shown. RHIP: Right hippocampus. b Representative [18]F-FDG-μPET 3D images showing FDG uptake of WT_Sed mice and AD_Sed mice at 10 months of age. c Quantification results of the standard uptakevalue (SUV) in the hippocampus between WT_Sed mice and AD_Sed mice at the ages of 10 months and 13 months. n = 10 at 10 months of age, n = 5 at 13 months of age. Paired t-test was applied between two groups. Data are shown as the mean ± SD, * p <0.05, ** p < 0.01, *** p < 0.001. [file 12974_2022_2401_MOESM1_ESM.tif]

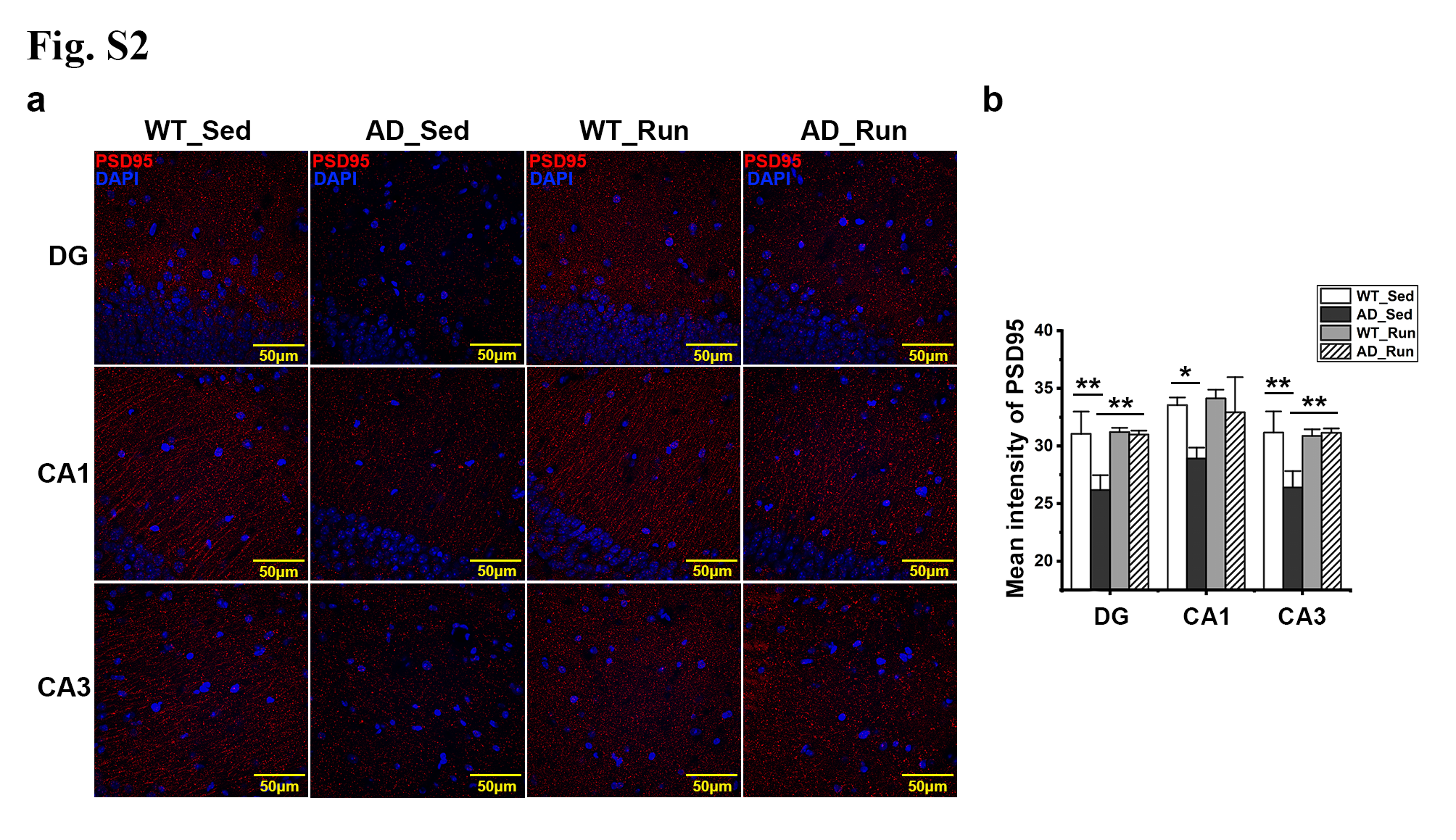

Supplement: Supplementary file 2 — Additional file 2: Figure S2. Running exercise enhanced the immunoactivity of PSD95 in the hippocampus of APP/PS1 mice. a Representative immunofluorescence staining of PSD95 (red) and DAPI (blue) in the DG, CA1, and CA3 regions of WT_Sed, AD_Sed, WT_Run, and AD_Run mice. Scale bar: 50 μm. b Quantification results of the mean intensity of PSD95 in the DG, CA1, and CA3 regions of WT_Sed, AD_Sed, WT_Run, and AD_Run mice. n = 3. Data are shown as the mean ± SD, * p < 0.05, ** p < 0.01,*** p < 0.001. [file 12974_2022_2401_MOESM2_ESM.tif]

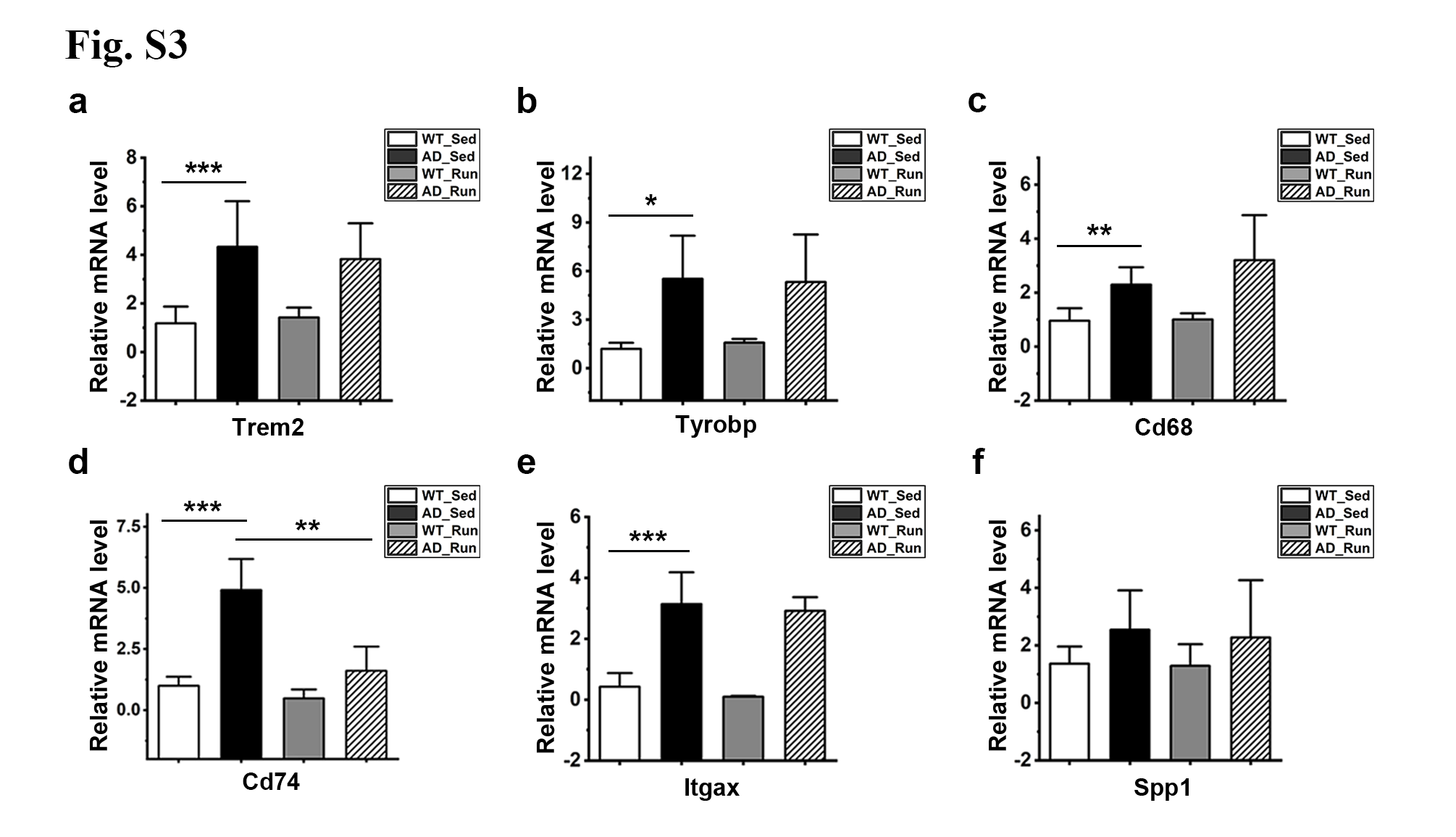

Supplement: Supplementary file 3 — Additional file 3: Figure S3. Relative mRNA levels of microglia-related genes in the hippocampus. a-f Quantification results of the relative mRNA levels of Trem2, Tyrobp, Cd68, Cd74, Itgax, and SPP1 in the hippocampus of WT_Sed, AD_Sed, WT_Run, and AD_Run mice. n = 3. Data are shown as the mean ± SD, * p <0.05, ** p < 0.01, *** p < 0.001. [file 12974_2022_2401_MOESM3_ESM.tif]
